# The Synthesis, Structure, Morphology Characterizations and Evolution Mechanisms of Nanosized Titanium Carbides and Their Further Applications

**DOI:** 10.3390/nano9081152

**Published:** 2019-08-11

**Authors:** Bai-Xin Dong, Feng Qiu, Qiang Li, Shi-Li Shu, Hong-Yu Yang, Qi-Chuan Jiang

**Affiliations:** 1 Key Laboratory of Automobile Materials, Ministry of Education and School of Materials Science and Engineering, Jilin University, Renmin Street NO. 5988, Changchun 130025, China; 2 Qingdao Automotive Research Institute of Jilin University, Qingdao 266000, China; 3 School of Mechanical and Aerospace Engineering, Jilin University, Changchun 130025, China; 4 National Demonstration Center for Experimental Materials Science and Engineering Education, Jiangsu University of Science and Technology, Zhenjiang 212003, China

**Keywords:** titanium carbides, nanostructures, excellent performances, crystal growth mechanism, morphology evolution manipulation

## Abstract

It is widely known that the special performances and extensive applications of the nanoscale materials are determined by their as-synthesized structures, especially their growth sizes and morphologies. Hereinto, titanium carbides, which show brilliant comprehensive properties, have attracted considerable attention from researchers. How to give full play to their potentials in the light-weight manufacture, microwave absorption, electromagnetic protection, energy conversion and catalyst areas has been widely studied. In this summarized article, the synthesis methods and mechanisms, corresponding growth morphologies of titanium carbides and their further applications were briefly reviewed and analyzed according to their different morphological dimensions, including one-dimensional nanostructures, two-dimensional nanosheets and three-dimensional nanoparticles. It is believed that through the investigation of the crystal structures, synthesis methods, growth mechanisms, and morphology characterizations of those titanium carbides, new lights could be shed on the regulation and control of the ceramic phase specific morphologies to meet with their excellent properties and applications. In addition, the corresponding development prospects and challenges of titanium carbides with various growth morphologies were also summarized.

## 1. Introduction

Titanium is a significant transition metal, and its carbide ceramics exhibit some superior comprehensive characteristics. Recently, the booming of titanium carbides ceramics has greatly promoted the advancement of light-weight manufacturing, microwave absorption, electromagnetic protection technology, energy conversion, and catalyzed synthesis, etc. [1,2,3,4,5,6] Throughout all of those development tendencies, titanium carbide ceramics almost always exhibit special morphology to follow the further applications. Actually, their prosperous applications can be well extended by controlling their extrinsic characteristics, especially sizes and growth morphologies.

As a matter of fact, with different synthesis reactants and methods, etc., various morphologies such as one-dimensional nanowires, nanorods, nanofibers, nanowhiskers and nanotubes; two-dimensional nanosheets; three-dimensional sphericities, octahedrons, truncated-octahedrons, cubes, hexagonal structures, dendrites, and terraces have been reported during the past several years, to name a few [7,8,9,10,11,12,13,14,15,16,17,18]. In addition, it is known that there are many conditions which will produce various morphologies of titanium carbides ceramics. In general, intrinsic factors such as the inherent crystal structure and thermodynamic properties including the surface energy and phase transformation entropy, as well as some external factors such as growth mechanisms, thermal and mass transportation in the melt will interplay with each other and form the final growth morphologies.

**Figure 1:**
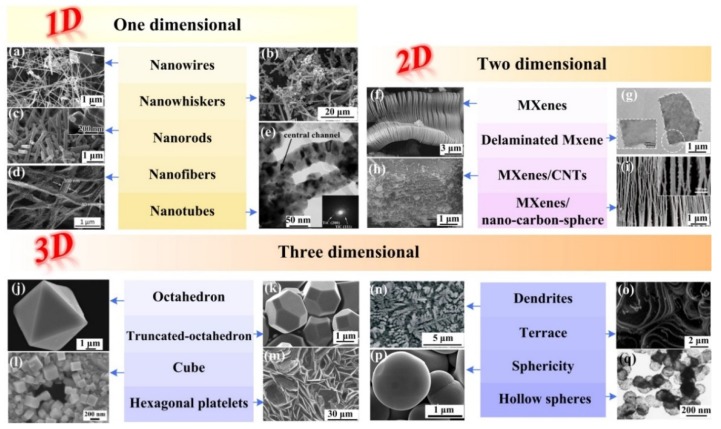
Various morphologies of titanium carbides with different dimensionalities reported recently and some of their applications. *One dimensional:* With permission from Reference [7], copyright (2013) Elsevier. With permission from Reference [8], copyright (2011) American Chemical Society. With permission from Reference [10], copyright (2015) Elsevier. With permission from Reference [22], copyright (2018) Elsevier. With permission from Reference [23], copyright (2010) Royal Society of Chemistry; *Two dimensional:* With permission from Reference [3], copyright (2018) Royal Society of Chemistry. With permission from Reference [12], copyright (2012) American Chemical Society. With permission from Reference [24], copyright (2019) American Chemical Society. With permission from Reference [25], copyright (2017) Royal Society of Chemistry; *Three dimensional:* With permission from Reference [13], copyright (2009) American Chemical Society. With permission from Reference [15], copyright (2012) Royal Society of Chemistry. With permission from Reference [16], copyright (2008) Elsevier. With permission from Reference [26], copyright (2017) the authors. With permission from Reference [17], copyright (2017) Royal Society of Chemistry. With permission from Reference [18], copyright (2011) Elsevier. With permission from Reference [27], copyright (2004) Elsevier.

**Figure 2:**
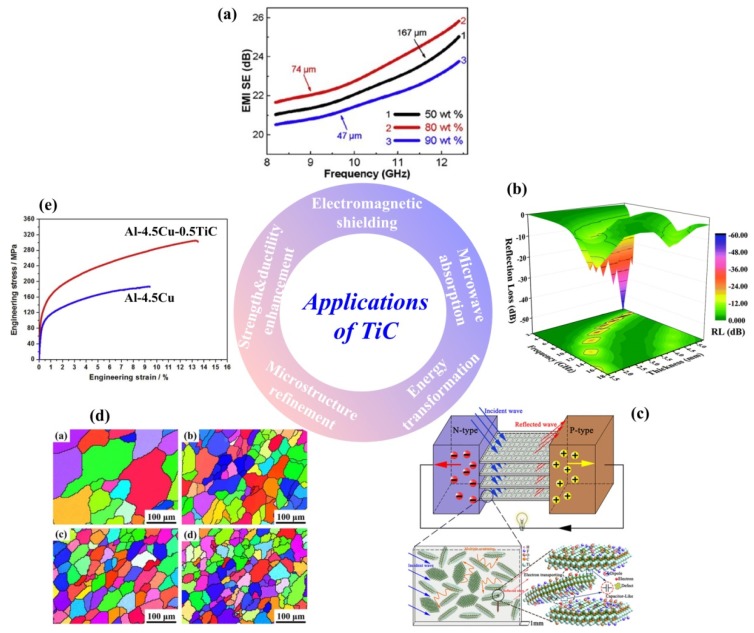
Various applications of titanium carbides nanostructures. (**a**) Electromagnetic shielding. With permission from Reference [28], copyright (2018) American Chemical Society. (**b**) Microwave absorption. With permission from Reference [3], copyright (2018) Royal Society of Chemistry. (**c**) Energy transformation. With permission from Reference [25], copyright (2010) Royal Society of Chemistry (**d**) Microstructure refinement. With permission from Reference [29], copyright (2018) Elsevier; (**e**) Strength&ductility enhancement. With permission from Reference [2], copyright (2018) Elsevier.

More potential titanium carbides nanomaterials, with at least one dimension between 1 nm to 100 nm, as restricted by the nanoscale, may display special characteristics such as size, shape, surface, interface, and structural effects [19,20,21]. Moreover, the characteristics of the above ceramics, such as the crystal sizes and morphologies, exposed facets and even surface roughness will bring significant differences in their physical and chemical properties. Due to the different growth behaviors and ultimately exposed surface, different dimensionalities of those ceramic will be synthesized. Correspondingly, some novel properties as well as resulting conceivable engineering and functional applications, such as in alloy refining and reinforcing, heat management, electromagnetic shielding, microwave absorption and chemical catalysis and energy transformation, just named a few areas, will be achieved. As exhibited in Figure 1 and Figure 2, the various morphologies mentioned before followed by diverse applications of different dimensionality titanium carbides have been summarized. Therefore, it is highly desirable to explore the growth regulation and mechanisms for titanium carbides ceramics synthesized through various preparation processes and to realize morphology control. The multiple dimensionalities of the growth morphologies of titanium carbide ceramics, which are controlled by the crystal growth processes, will be detailed and summarized in the following chapters.

In this review, the synthesis, growth and corresponding morphologies of titanium carbides and their further applications are summarized. The morphology characterizations and evolution mechanisms of titanium carbides have been systematically expatiated. Finally, the prospects for development and some potential research orientations in the future are also put forward.

## 2. The Crystal Structure and the Physical and Chemical Properties of Titanium Carbides

Titanium carbides have been highly attractive to researchers from a theoretical and fabrication point of view in the past decades. Particularly, distinguished by a high hardness (28~35 GPa), high specific strength, high Young’s modulus (300~480 GPa), relatively low density (4.92 g/cm^3^), high melting point (3067~3340 °C), high thermal conductivity, and low coefficient of thermal expansion (6.4 × 10^−6^/°C), as well as good wear and corrosion resistance, titanium carbides have shown splendid prospects. [2,14,15,16,19,21,29,30,31] In the past few years, many researchers have concentrated on exploring the relationship between the physical and chemical properties of titanium carbides and their sizes, morphologies, compositions and structures, which will shed new lights on the applications in various industries [7,8,9,10,11,12,13,14,15,16,17,18,22,23,24,25,26,27,28].

### 2.1. The Crystal Structure of Titanium Carbides

**Figure 3:**
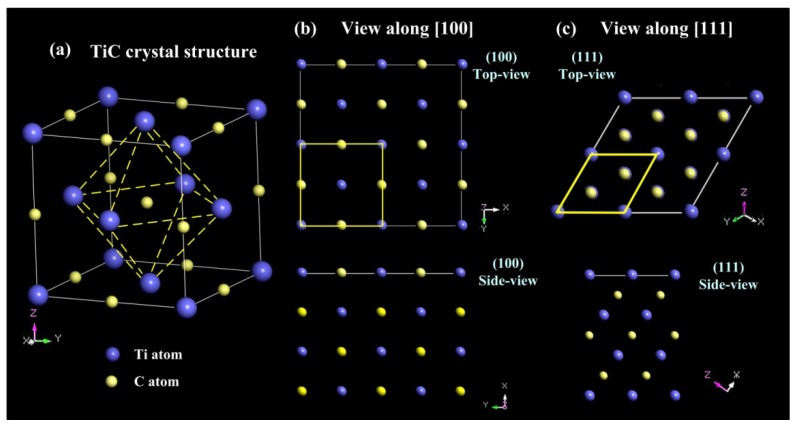
(**a**) The crystal structure of TiC; (**b**) The TiC crystal structure view along [100]; (**c**) The TiC crystal structure view along [111].

Generally, titanium carbides (TiC) are face-centered cubic crystal NaCl-type structure (FCC) with space group Fm-3m (225). As shown in Figure 3a, Ti atoms occupy the position of each corner and each face centers in the cubic structure, while nonmetal C atoms occupying the center position of each edge. The dominating low-index surfaces in face-centered cubic structures are mainly (100), (110) and (111). It is known that (100) and (111) planes are the most stable crystal planes for FCC crystal [30,32]. Remarkably, Ti and C atoms exist simultaneously in TiC (100) planes, as presented in Figure 3b. When viewed along the [111] direction, C and Ti atoms show an alternate arrangement between each stacking layer. Therefore, such layered alternate stacking of C and Ti atoms will give the TiC (111) planes intense polarity.

**Figure 4:**
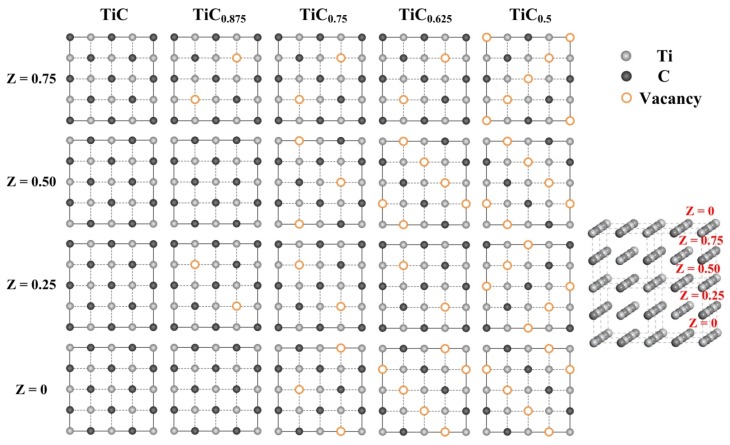
The structures schematic diagrams of TiC*_x_* phases with different stoichiometric ratio (as calculated by a 16-atom supercell). Z shows the different planes in the TiC*_x_* crystal, and the four planes from top to bottom are marked as 0, 0.25, 0.5 and 0.75, respectively.

In another point of view, titanium carbides can be seen as a solid solution with C solute atoms dissolved into the Ti cubic crystal cells, and C atoms will occupy the interstitial spaces between Ti atoms. Therefore, it can be predicted that C atoms, with their smaller atomic radius, will be easily moved from their original sites. Actually, due to relatively high synthesis temperature and slow C atoms diffusion rate in the melt, a large number of C vacancies will appear in the titanium carbides (TiC*_x_*) structure and give rise to substoichiometric defects. Those vacancies of C will strongly affect the actual C/Ti ratio of titanium carbides (TiC*_x_*). Many studies have found that a full stoichiometric ratio TiC is hard to obtain. The stoichiometric ratio *x* of TiC*_x_* can vary from 0.48 to 0.98 and is stable within a wide stoichiometric ratio [33,34]. The C vacancies also influence the TiC*_x_* crystal size, morphology and even crystal structure. It is known that under the same conformations (as produced by consistent reaction and environment conditions), with the carbon vacancy concentration decreasing, the defective surfaces will become more stable [35]. Although the appearances of the vacancies are random, the positions of C vacancies can be well predicted and applied in the further first-principle calculations, which focus on the surface energy or density of states, and some other properties that arise upon forming an interface with another metal or ceramic can also be calculated, such as the interface energy and work of interfacial adhesion and separation. Based on the theory of Hugossonand [36], it is more favorable in terms of energy to set two Ti atoms with two nearest-neighbor C vacancies compared with the case of one Ti atoms with one or three C vacancies. C vacancies with the stoichiometric ratio *x* (0.5, 0.625, 0.75, 0.875 and 1.0) as well as the atom stacking layers are illustrated in Figure 4.

### 2.2. The Characteristics of the Chemical Bonds in TiC_x_

According to the atomic composition and the crystal structure of TiC*_x_*, the chemical bonds are also worth considering. The simultaneous existence of ionic bonds, covalent bond and metallic bonds [37] will bring titanium carbides intriguing characteristics. It has been indicated that the metallic bonds come from Ti-Ti bonding and the covalent bonds come from the interaction between the C-2s and C-2p orbitals with Ti-d orbitals. Charge transfer between Ti and C atoms may account for the ionic bonds. In transition metal carbides, the 2p orbitals in carbon are close in energy to the metal 3d orbitals. Yang et al. [38] utilized the first-principle calculation to evaluate the mechanical properties of TiN and TiC. They found that the hybridization between Ti-3d and C-2p or N-2p electrons may account for the ultra-hardness of TiN and TiC. Additionally, the considerably stronger π-bonding in titanium carbides will overcome the loss of exchange energy, giving TiC*_x_* a much stronger bond, which is approximately 3.857 eV [39].

Moreover, the corresponding crystal planes are also dominated by the chemical bond, which concretely reflects on the properties of different crystal planes. Due to the following advantages, the TiC (100) surface is chosen as the substrate to catalyze the reduction of NO by Chu et al. First, (100) surface of TiC has stable termination; then, after full relaxation, the TiC (100) surface does not reconstruct, and in addition, the (100) surface relaxation is not large. In this study, a Pt monolayer is supported on a TiC (100) substrate to form an efficient catalyst (Pt/TiC) [6]. Back and Jung investigated the catalytic properties of bare TiC (100) surfaces and found that the catalytic activity of TiC is expected to be active and selective for CO_2_ reduction to CH_4_ [40]. Wang et al. found that Li_2_O_2_ could adsorb and deposit on the TiC (111) surfaces and their study also demonstrated that it is feasible to use TiC as a cathode material for Li air batteries [32]. It is established that for cubic TiC, the (100) surface are able to absorb several different molecules including water, methanol, ethanol, NH_3_ and CO_2_, etc., and the (111) surfaces are highly active in the dissociative adsorption of hydrogen [31,41]. Moreover, the adsorption and dissociation processes of O_2_ on the TiC (100) surface have also been reported [42].

### 2.3. The Relationship between the Crystal Structures and the Growth Morphologies of Titanium Carbides

For face-centered cubic crystals, (100) and (111) are the most significant low index facets. Generally, Wulff’s theorem can give us a direction to predict the equilibrium morphology of a crystal [43]. It is known that the close-packed planes characteristic of high reticular densities and large interplanar spacings will present lower surface energies, and planes with low surface energies are more stable and become the exposed crystal planes finally. Moreover, studies based on Wulff’s theorem have been widely reported, and substantial progresses have also been made concerning the equilibrium crystal growth theory since the 1980s. The studies about equilibrium crystal growth morphology have usually concentrated on the cusps, facets, sharp edges, forbidden regions, surface reconstructed and surface adsorption of the crystal or used Wulff plots to construct and forecast some crystal structures. Moreover, the equilibrium growth of the nanostructures is complicated, and this is because the morphology of a nanoscale crystal is sensitive to the changes of atoms. The addition or removal of a single atom will lead to a substantial change in the crystal morphology [44].

**Figure 5:**
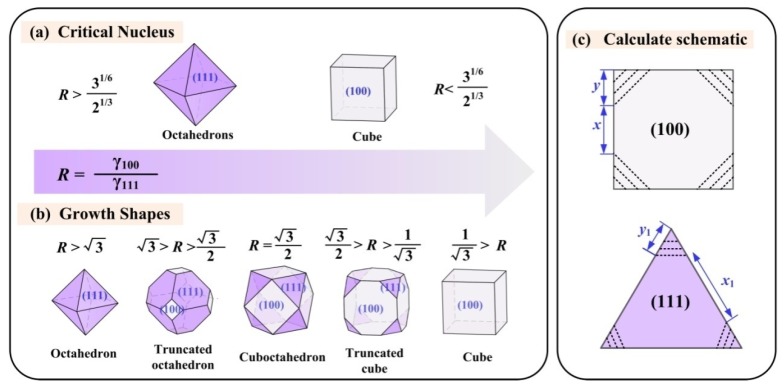
The surface energy ratio limits for observing the (**a**) critical nucleus and (**b**) growth shapes in the cuboctahedral morphologies, (**c**) The calculated model of octahedron and cube.

Figure 5a,b shows the morphology transformations of FCC ferrite nanoparticles, and the nucleation and growth models were investigated by Swaminathan et al. The ferrite nanoparticles in their studies were faceted-cubic, cuboctahedral polygonal structure with mainly (100) and (111) typical faces. They suggested that the ratio of the surface energies *R* (γ100/γ111) will restrict the critical nucleation and growth morphologies [45]. They calculated the Helmholtz free energy of the faceted nanoparticles and determined the critical nucleus shape according to both the first and second derivatives of the Helmholtz free energy with respect to the parameter *x* (*x*_1_) and *y* (*y*_1_), and the models are shown in Figure 5c. The results illustrated in Figure 5 show the specific relationship between the surface energy ratio (*R*) and the critical nucleus and growth shapes, and those findings will give rise to a visualized way to estimate the final morphologies of growth phases.

In general, for single phase metal crystal, (111) planes are the most close-packed planes, which should have the lowest surface energy and be the most stable. Moreover, Zhang et al. calculated the surface energies of (111), (100) and (110) planes in a face-centered cubic crystal, and their results suggested that the surface energies were in the following order: γ111 < γ100 < γ110 [46]. However, according to the TiC*_x_* crystal structure, there exists a relatively high divergent electrostatic energy on the (111) surfaces of TiC*_x_*, which give them theoretically high activities [47]. It was determined by Ilyasov et al. that the chemical activity of the (111) surface is higher than that of (100) surface by considering the influence of vacancies on the electronic and structural properties of TiC (100) and (111) planes [48]. Therefore, it seems that the stability and other properties of (100) and (111) planes in TiC*_x_* cannot be estimated in the same way used for metallic FCC crystals. More reaction conditions, synthetic methods, preparation processes and reactant compositions should be considered which will influence the properties of specific crystal planes.

In theory, because of a high fusion entropy ∆S_m_ (which is approximately 21 J/Kmol) as well as a large Jackson alpha factor a_J_ (approximately 5~7), the growth mode of titanium carbide is typical faceted growth [14]. In addition, under different reaction conditions, the growth morphologies will be varied, such as cubes, truncated octahedrons, sphericities, dendrites, terraces and even different dimensional morphologies can be obtained. Therefore, it can be seen that the crystal structure is not the only factor that controls the growth behaviors. Both intrinsic and external conditions should be considered in this case to forecast and control the growth of titanium carbides.

According to the above findings, the electronic structure and chemical bond, etc. will influence the growth behaviors from the intrinsic perspective and the crystal will grow into equilibrium morphology. However, some external conditions including the reaction process, solute transportation process and interaction interface, reaction system and reaction temperature, impurity doping, substrate and catalyst, just to name a few, will strongly influence the growth behaviors of ceramic phases. It can be seen that under some conditions, such as self-propagating high temperature synthesis, the reaction is nonequilibrium and the morphologies of the products may be diverse. It is obvious that various external morphologies nanostructures, such as one-dimensional nanostructures, two-dimensional nanostructures (the crystal structure may not remain face-centered cubic) and even three-dimensional nanostructures have been widely reported over the years. Additionally, it can be predicted that particular growth morphologies will take on some special application functions. For instance, when utilized in alloying systems, the control of specific exposed surfaces of TiC*_x_* could reduce the lattice mismatching between the matrix alloy and titanium carbide ceramics, which can be used to realize engineering fabrications such as solidification structures regulation and alloying reinforcement. Moreover, such selective exposed surfaces can well achieve chemical catalysis. Some one-dimensional nanostructures can not only reinforce the matrix but can also effectively absorb microwave via specific surfaces. In addition, two-dimensional nanostructures have more similar functions. In summary, those abundant morphology characteristics of titanium carbide ceramics will create more opportunities to broaden their application areas and make them good candidates for achieving their excellent value in alloy refinement and reinforcement, electromagnetic shielding, microwave absorption, heat management, and chemical catalysis.

## 3. The Synthesis and Characterization of One-Dimensional Titanium Carbides as Well as Their Applications

### 3.1. The Growth of Titanium Carbides during the Chemical Synthesis

Recently, plenty of interest in one-dimensional (1D) nanoscale titanium carbide ceramic materials, such as nanorods, nanowires, nanowhiskers, nanotubes, and nanofibers has been stimulated; especially in the fields of the structural component reinforcement, microwave absorption and catalysis. Those extensive applications may be due to the unique physical and chemical properties imbued by special crystal morphologies. To date, many techniques including the biotemplate method, (chloride-assisted) carbothermal reduction, chemical vapor deposition and electrospinning [7,8,10,49,50,51,52,53] have been reported to fabricate 1D TiC nanostructures. Additionally, various carbide sources play important roles in the reaction system and have attract much attention from researchers. In addition to some conventional carbide source such as carbon black, carbon nanotubes [9], other novel carbide sources such as cotton T-shirt, phenolic resol, microcrystalline cellulose, sucrose and polyvinylpyrrolidone have also been introduced into the reaction system to obtain TiC with various morphologies [7,8,10,49,50].

**Figure 6:**
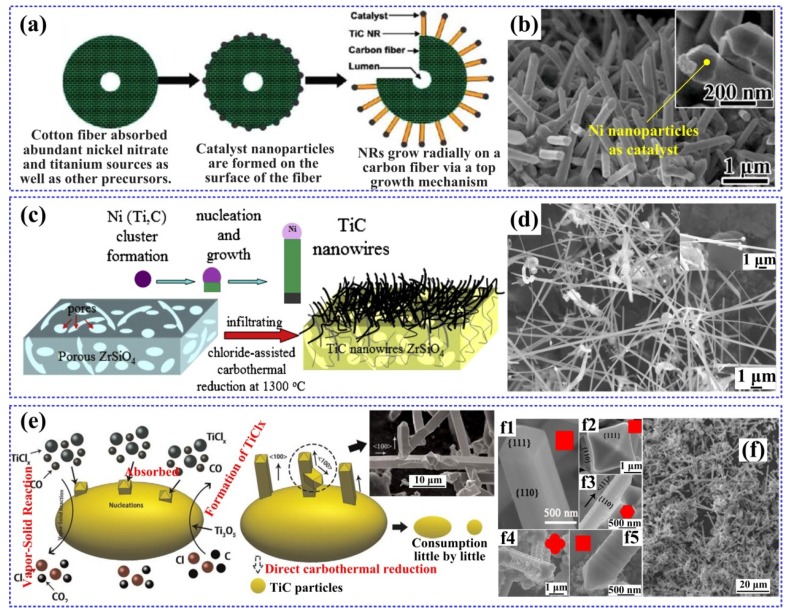
(**a**) The cross-sectional schematics of the TiC nanorods formation mechanism and (**b**) a high-magnification SEM image of nanorod arrays along the cotton fiber radial direction. The inset of b shows the catalyst Ni particles on the tips of the nanorods, with permission from Reference [8], copyright (2011) American Chemical Society; (**c**) Synthetic approach for TiC nanowires-ZrSiO_4_ and VSL growth of TiC nanowire and (**d**) the as-prepared TiC-ZrSiO_4_ composites. The high-magnification image of TiC nanowires is shown in the inset with permission from Reference [7], copyright (2014) Elsevier. (**e**) A simple mode for the VS growth of TiC whiskers on Ti_3_O_5_ particle. The left image is the nucleation of TiC on a Ti_3_O_5_ particle. The right image shows faceted TiC whiskers growing along the [100] direction, with the TiC epitaxially growing into a branch structure on the lateral surface of a TiC whisker shows in the inset (**f**) The cross section of TiC whiskers and SEM images of the TiC whiskers after separation with permission from Reference [10], copyright (2015) Elsevier.

Tao et al. successfully synthesized a kind of single-crystalline TiC nanorods via a cost-effective and facile biotemplate method [8]. Natural nanoporous cotton fibers from the commercial cotton T-shirt, which acted as both the carbon source and the template, were used to simplify the synthesis process of TiC nanorods. The radial growing of abundant straight TiC nanorods on the entire length of the carbon microfiber was obtained, and the sizes of the as-synthesized nanorods were approximately 80–200 nm in diameter and 1–3 μm in length. This result revealed the synthesis mechanism of these TiC nanorods, as shown in Figure 6a, which can be seen as a chloride-assisted vapor-liquid-solid (VLS) growth mechanism with a Ni nanoparticle as a catalyst at the tip of each nanorod. Figure 6b shows an image of the TiC nanorods with the Ni catalyst. The researchers also measured a high Young’s modulus of the TiC nanorods, which will provide them splendid application prospects in nanoelectromechanical systems as structural/functional building blocks or as significant reinforcements for some composites.

Titanium carbide (TiC) nanowires were also fabricated by Yuan et al. via infiltrating and subsequently chloride-assisted carbothermal reduction on the porous ZrSiO_4_ ceramic substrate [7], as presented in Figure 6c. The TiC nanowires grew along the direction of the [001] zone axis, and their final morphology were approximately 300 nm in diameter and up to several microns in length, as Figure 6d shown. Similar catalyst-assisted growth mechanism can be seen in this study, wherein the nanoclusters of Ni (Ti, C) acted as the nucleation sites. They also considered that the porous substrate structure will improve the adhesive ability, reduce the contact resistance and ensure easy electron transportation. Following the Fowler-Nordheim behavior, the field emission properties of TiC-ZrSiO_4_ as cathodes in this study exhibited a low turn-on field which was approximately V/μm, hence, it can play a significant part in the application of field emission. In another work by Yuan et al. [49], they synthesized TiC nanowires by the same chloride-assisted carbothermal reaction process using sucrose as the carbide source. In this case, TiC nanowires with high specific surface area could be obtained (186.7 m^2^ g^−1^), which were approximately 200–400 nm in diameter and had lengths of about dozens of micrometers. Remarkably, the TiC nanowires/paraffin mixture showed prominent electromagnetic wave absorption ability.

Xiong et al. [10] mainly confirmed a vapor-solid (VS) mechanism for the epitaxial growth of TiC whiskers on Ti_3_O_5_ particles. Without a catalyst, the faceted TiC whiskers grew into different morphologies under the effects of different carbon sources (chars, microcrystalline cellulose and pyrolytic carbon black), which are shown in Figure 6(f1–f5). This phenomenon may be due to changes in the corresponding supersaturation of the vapor phases (TiCl_x_ and CO) with the variation of carbon sources. As Figure 6e demonstrated, after the formation of the TiC nucleus on Ti_3_O_5_ particle, the growth adatoms will persistently absorb and deposit on (111) faces at the tip, which can lead to the TiC nucleus transform into TiC whisker. It is known that the formation of the 2D nuclei needs a high reactant concentration [13]. Also, the decomposition degrees of different carbon sources are different, and during the heating process, more carbon gases will be produced by the decomposition of cellulose. As the vapor phase supersaturation increasing, TiC whisker showed a circular cross-section firstly. Then through 2D nucleation on the templates of Ti_3_O_5_, the TiC whiskers transformed into a regular hexagon or a faceted square cross section. Especially, the cross section of the TiC whiskers from pyrolytic carbon black as carbon source exhibited dendritical cross-section with the lateral growth along the [100] direction.

In summary, the synthesis of 1D nanostructures by vapor-phase growth methods is mainly based on two mechanisms: one is vapor-solid (VS) and vapor-liquid-solid (VLS). In addition, whether or not there exists a catalyst at the top of the nanostructure is the main distinction between the two growth mechanisms. It can be seen that the vapor-liquid-solid (VLS) growth mechanisms always need a catalyst like Ni or its compounds. Additionally, it can be predicted that some novel carbon sources with large sizes will deposit during heating and will significantly affect the TiC morphology.

The discovery of carbon nanotubes (CNTs) in 1991 promoted the development of new one-dimensional (1D) nanostructured materials for potential applications [50]. In addition, some studies suggested that these one-dimensional TiC will change their nanostructures with the variation of the reactants concentrations during the reaction process. Taguchi et al. [11] utilized the reaction between carbon nanotube (CNTs) aggregates and Ti vapor at approximately 1300 °C to fabricate TiC nanostructures. In this case, Ti existed as vapor and was progressively consumed on the surface of the CNTs aggregates. They also demonstrated that the Ti vapor content will affect the morphology of the as-synthesized TiC. As the depth below the surface of the CNTs aggregates increased, three different TiC nanostructures were formed in sequence: TiC nanowires, TiC nanotubes, and CNTs decorated with TiC nanoparticles on their surfaces. Additionally, it can be observed that some single-crystalline TiC nanotubes with lengths approximately 300 nm grew along the [110] direction. Saba used a novel pressureless spark plasma sintering (SPS) technique and successfully produced TiC-modified carbon nanotubes, TiC nanotubes and TiC nanorods using CNTs and Ti powder [22]. Similar to Taguchi, they found that the morphologies of the synthesized nanostructures depended strongly on the Ti concentration.

### 3.2. The Electrospinning Technique to Synthesize TiC/C Hybrid Nanomaterials

Recently, the electrospinning technique has been repeatedly reported as an innovative functional technology with great potential, which is also a simple but low-cost method to produce 1D nanostructures especially nanofibers. When produced via the electrospinning technique, nanofibers usually exhibit structures in the range of nanometer to a few micrometers [51]. Compared with above 1D nanostructures, nanofibers have some attractive characteristics to be used in some energy storage systems. By the electrospinning method, isotropically conductive TiC/C hybrid nanofibers have been prepared by Ren et al. [52] The morphology of the as-produced TiC/C hybrid nanofiber is uniform with an average diameter about 100 nm. In a typical process, dimethylformamide was dissolved by polyvinylpyrrolidone and acetic acid to form the transparent solution. Tetrabutyl titanate was dropwise incorporated into the above solution to obtain a transparent solution for further electrospinning processing. Compared with pure TiC nanoparticles and carbon materials with similar specific surface areas reported previously, the resulting TiC/C hybrid nanofibers showed a much higher specific capacitance. Therefore, these TiC/C hybrid nanofibers can be regarded as promising candidates to applicate to supercapacitor. Submicron-scale titanium carbide-carbon (TiC-C) hybrid nanofibers have been fabricated by Cho et al. via electrospinning as well as a carbothermal reduction reaction [53]. They found an increasing in electrical conductivity under an elevated temperature of carbothermal reduction as well as the addition of TiC to the carbon fibers. Similar TiC-C nanofibers structures made by the electrospinning method have been reported to more various applications. Zhou et al. also utilized the electrospinning method to synthesize titanium carbide—carbon nanofibers (TiC/CNF) and found distinguished mechanical and electrical properties [54]. Also, such nanofibers (TiC/CNF) are promising to find their applications in titanium matrix composites (TMC), electronic devices as well as efficient catalyst scaffolds. Table 1 summarized some 1D titanium carbides and their corresponding sizes, morphologies, preparation methods, carbon sources and application areas. In addition, by controlling the specific fabrication processes, especially controlling the external factors mentioned in the first chapter during the growth of those 1D nanostructures, various sizes and morphologies can be realized. Obviously, it can be seen that those special morphologies of titanium carbides can give them more preferable characteristics for diverse applications.

## 4. The Synthesis and Characterization of Two-Dimensional Titanium Carbides, as Well as Their Applications

Compared with one-dimensional (1D) nanomaterials extended in one direction, two-dimensional (2D) free-standing crystals have tended to be more prevalent in the past several years. Inspired by graphene and other layered materials, which can be exfoliated into 2D sheets, the 2D titanium carbides with high aspect ratios and thicknesses within several atomic layers have also been reported recently, and some researchers have vividly described that those 2D titanium carbides exhibit accordion layered structures. Such layered structures can endow 2D titanium carbides with intriguing peculiarities, such as a high relative complex permittivity, strong microwave attenuation ability, excellent EMI shielding properties, high catalytic activity, etc., which make 2D titanium carbides competitive and promising materials for use in electronic devices, supercapacitors, catalysis, microwave absorption, EMI shielding areas and energy conversion. [3,4,5,55,56] In this chapter, the synthesis methods, synthesis mechanisms, comprehensive properties and applications of different morphologies of two-dimensional titanium carbides are briefly summarized.

### 4.1. The Fabrication of Layered Precursor MAX Phases

**Figure 7:**
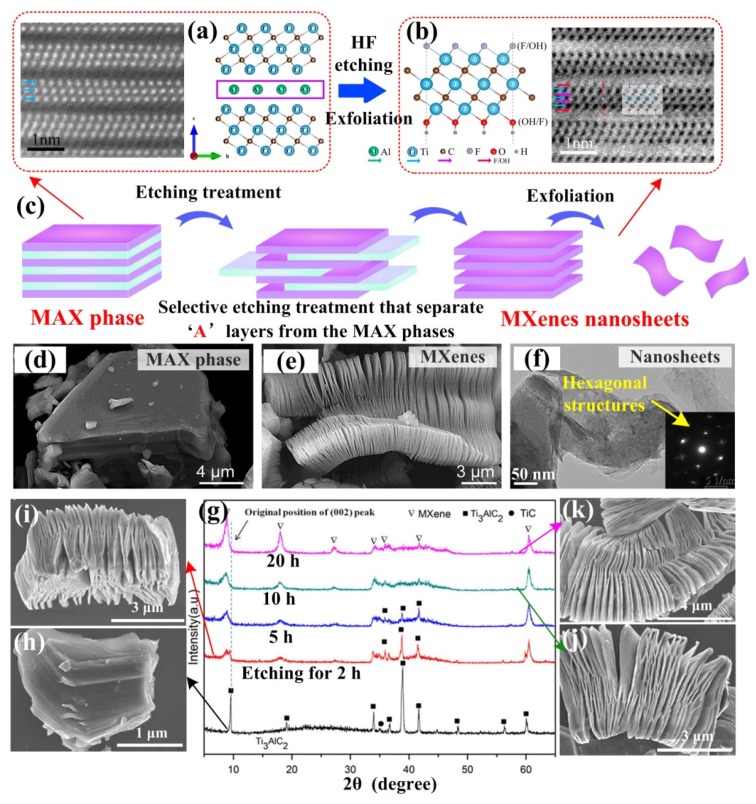
(**a**) The atomic structure of the Ti_3_AlC_2_ MAX phase and a high-angle annular dark-field (HAADF) image of multilayer Ti_3_C_2_T_x_ (here the image only shows the inheritance of Ti_3_C_2_T_x_ from the parent Ti_3_AlC_2_, so we use this image to represent the MAX phases arrangement, and the inherited Ti_3_C_2_T_x_ can be seen as a Ti(s)-C-Ti(c)-C-Ti(s) arrangement). The final Ti_3_C_2_T_x_ monolayers in (**b**) show that on both sides of the Ti_3_C_2_ layer, the functional groups (-O and/or -F) atoms favor staying on top of the Ti(c) atoms rather than at the topmost sites of the C atoms, with permission from Reference [64], Copyright (2015) American Chemical Society. (**c**) The schematic diagram of the etching treatment and exfoliation process to remove the ‘A’ layer from the MAX phase to obtain MXenes. (**d**–**f**) The actual morphology evolutions from the MAX phase to MXenes, and to nanosheets by exfoliation, with permission from Reference [12], copyright (2012) American Chemical Society. With permission from Reference [62], copyright (2015) Elsevier. (**g**) The X-Ray Diffraction (XRD) analysis of Ti_3_AlC_2_ before and after HF treatment for 2 h, 10 h, and 20 h. (**h**–**k**) are the corresponding SEM images of the as-prepared MXenes. With permission from Reference [66], copyright (2016) Elsevier.

In general, titanium carbides that come from a family of 2D transition metal carbides “MXenes” are fabricated by the selective extraction of specific atoms from their layered precursor “MAX” phases. The MAX phases are ternary carbides and nitrides denoted by the M_n+1_AX_n_ formula (*n* = 1, 2 or 3). Here, ‘M’ represents early transition metal elements such as Ti, ‘A’ mainly represents group IIIA or IVA elements such as Al and Si, while ‘X’ is C or N atoms, as Figure 7a shown. Ti_3_AlC_2_ and Ti_2_AlC are representative compounds. MAX phases are layered hexagonal crystal structures with a P6_3_/mmc space group. Under normal conditions, overall MAX phases are chemically stable; however, the ‘A’ layers are relatively chemically active due to the weaker A-X and A-M bonding compared to M-X bonding [57], so it can be speculated that the ‘A’ atom layers are easy to remove the MAX phases, after which the bulk structures (M_n+1_AX_n_) will turn into accordion layered structures (M_n+1_X_n_). In addition, common 2D titanium carbides are Ti_3_C_2_ and Ti_2_C.

Shahin et al. synthesized the Ti_3_AlC_2_ MAX phase by a mechanochemical synthesis method in a Ti-Al-C reaction system [58]. The initial mixtures of Ti, Al and C were produced according to the stoichiometric ratio of Ti_3_AlC_2_ (Ti:Al:C = 3:1:2), and those mixtures were milled at a rotation speed of 450 rpm for up to 10 h. The products in their study were mainly Ti_3_AlC_2_ and TiC. And compared with the MAX phase, the TiC phase was easier to form the environment of a high temperature melt. This is because more liquid phases existed, which could promote the synthesis of TiC. Purer Ti_2_AlC has also been synthesized by sonochemical combustion synthesis in Al-Ti-C system. Liu et al. [59] used Ti-Al-C reactants (molar ratio of Ti:Al:C = 2:1:1) and found that after ultrasonic treatment, Ti_2_AlC was the main products while Ti_3_AlC_2_ was the main products without ultrasonic treatment. The MAX phases can show some special characteristics and can be applied to the alloys to improve their comprehensive properties. Yu et al. found that Ti_2_AlC MAX phases can be excellent reinforcements for AZ91D Mg alloy, especially enhancing the wear resistance properties and self-lubrication ability [60].

### 4.2. The Synthesis Processes of “MXenes” from MAX phases

Naguib’s work published in 2011, firstly put forward the concept of “MXenes”, which is the exfoliation product from Ti_3_AlC_2_ parent phase and is regarded as a new kind of 2D materials [12,61]. The name “MXenes” proposed by Naguib mainly emphasized the similarity of these 2D solids to graphene. The MAX phase in this study was Ti_3_AlC_2_, and the extraction of the Al layers from the Ti_3_AlC_2_ was realized by an exfoliation process using hydrofluoric acid (HF). The specific reaction processes simplified as Equations (1) to (3) show the reaction between the MAX phase Ti_3_AlC_2_ and hydrofluoric acid after the Ti_3_AlC_2_ phase was immersed in hydrofluoric acid:

Ti_3_AlC_2_ + 3HF = AlF_3_ + 3/2H_2_ + Ti_3_C_2_ (1)

Ti_3_C_2_ + 2H_2_O = Ti_3_C_2_(OH)_2_ + H_2_ (2)

Ti_3_C_2_ + 2HF = Ti_3_C_2_F_2_ + H_2_ (3)

From the crystal structure illustrated in Figure 7b, it is obvious that after the exfoliation of Al, the 2D Ti_3_C_2_ layers exhibited two exposed Ti atoms per unit formula and will be satisfied by the -OH and/or -F surface functional groups (which were the most likely ligands groups due to the aqueous environment enriched in fluorine ions during the synthesis procedure). The complete process of the synthesis of MXenes nanosheets, which includes etching and exfoliation, is vividly shown in Figure 7c, and Figure 7d,e exhibit the SEM images of Ti_3_AlC_2_ phase and Ti_3_C_2_, respectively. It can be seen from Naguib’s work in Table 2 that the etching process did not change the hexagonal crystal structure but did change the unit cell parameters of the as-prepared crystals. Meanwhile, the unit cell volume also increased when the functional group was attached to the terminated surface. The illustration of Figure 7f also exhibits that the Ti_2_C nanosheets retained hexagonal crystal structure after etching treatments [62]. Sometimes, some -O groups also exist, which may result from etching at a low concentration of HF, and MXenes processing smaller specific surfaces and higher proportions of -O surface groups will appear [63].

It is known that MXenes are virtually a class of 2D inorganic compounds, they combine metallic conductivity (from transition metal carbides) and a hydrophilic nature (due to their -OH or -O terminated surfaces, when the terminated surface functional groups exist). To date, many methods have been reported to obtain 2D MXenes, but by etching-assisted exfoliations from MAX phases are considered to be the main method to produce the massive monolayer and multilayer MXenes currently. This etching-assisted exfoliation process can also be effectively accelerated by ultrasonic treatment, among others [64,65]. Moreover, because the synthesis of MXenes relies on the process of etching, many factors will influence the final morphologies of 2D titanium carbides MXenes, including different etching solutions, diverse etching times, different etching temperature, the time and temperature of heat treatment after etching, etc. [66,67,68] Wang et al. found that with increasing etching times, the strongest (104) peak gradually weakened and disappeared, and the morphology of the stacked sheets become thinner with further delamination [66]. Sun et al. found that higher etching temperature will lead to much faster transformation processes from Ti_3_AlC_2_ to Ti_3_C_2_ MXenes [68].

As mentioned above, the removal of ‘A’ atoms layers from MAX phases are always realized by etching processes. Therefore, the surfaces of the as-synthesized MXenes are easily terminated with other functional groups, such as -F, -OH/-O, and the MXenes are also named M_n+1_X_n_T_x_ (abbreviated as M_n+1_X_n)._ Etching-assisted exfoliation is promoted not only by HF etching, but also by various fluoride salts in hydrochloric acid including LiF, NaF, KF and NH_4_F, which can also achieve the desired effect [69,70]. Liu et al. found that different positive ions (such as Li^+^, Na^+^, K^+^, and NH_4_^+^) in HCl solutions will make different surface structures of as-prepared MXenes, which will finally affect the methane adsorption properties of those MXenes [69]. Furthermore, considering that a high concentration of HF can contaminate the environment and is very dangerous and harmful to researchers during the etching, a safer and more effective method to produce 2D MXene has been reported by Feng et al. By exfoliating Ti_3_AlC_2_ using bifluorides (NaHF_2_, NH_4_HF_2_ and KHF_2_) in lieu of HF, a larger interplanar spacing of Ti_3_C_2_ can be obtained by a single-stage process [71].

### 4.3. More Promising Function Applications of 2D MXences

As mentioned above, it can be seen that the special internal multilayer structure and the terminated functional groups will bring more specific characteristics to 2D titanium carbides. For the terminated group of ‘T’ (T represents -F and -OH/-O) in M_n+1_X_n_T_x_, Wang et al. found that heat treatment can effectively eliminate the -OH and -F groups on the surface of the Ti_3_C_2_ nanosheets to obtain bare MXenes [66]. More importantly, they presented that the bare MXenes performed better in term of electrical properties compared with the MXenes surface functionalized by -OH and -F. Tang suggested that the metallic characteristic (mainly of bare Ti_3_C_2_ nanosheets) or narrow-band gap semiconducting characteristic (mainly of Ti_3_C_2_ with -OH/-F terminated surface functional groups) will give rise to applications of Ti_3_C_2_ nanosheets in Li^+^ batteries [57]. Combining excellent comprehensive properties such as a low diffusion barrier and open circuit voltage with good electrical conductivity and high theoretical Li capacity, bare Ti_3_C_2_ nanosheets can be good candidates for use as anode materials to replace TiO_2_ in Li^+^ batteries. For -F/-OH terminated two-dimensional Ti_3_C_2_, such surface functionalization tends to decrease the diffusion of Li while decreasing the storage capacity of Li, so these phenomena are not favorable to the practical synthetic and applications in batteries. Therefore, it can be seen that by designing and regulating 2D Ti_3_C_2_ nanosheets, insightful applications as electronic and energy storage materials are provided with bright prospects.

Shahzad et al. utilized Ti_3_AlC_2_ and LiF to synthesize delaminated Ti_3_C_2_T_x_ without ultrasonic treatment and used the productions to fabricate Ti_3_C_2_T_x_-sodium alginate composites [72]. Those Ti_3_C_2_T_x_-sodium alginate composites showed excellent EMI shielding effectiveness: the 0.045 mm Ti_3_C_2_T_x_ film and 0.008 mm Ti_3_C_2_T_x_-sodium alginate (which contained 90 wt.% Ti_3_C_2_T_x_) produced electromagnetic interference shielding effectiveness at 92 dB and 57 dB, respectively. The excellent electrical conductivity and the effects of multiple internal reflections can account for the excellent EMI shielding performance of Ti_3_C_2_T_x_ composites.

**Figure 8:**
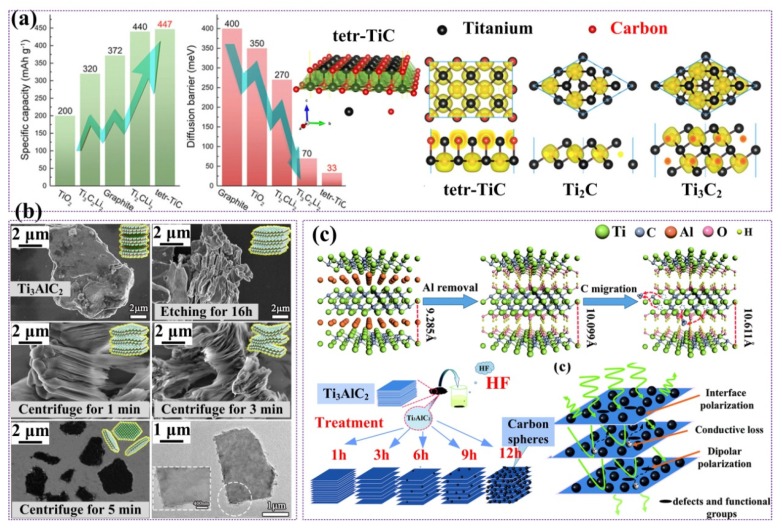
(**a**) The optimized geometries of the free-standing tetr-TiC sheet and the comparison of different titanium carbide materials, with permission from Reference [5], copyright (2018) American Chemical Society. (**b**) The morphological evolution of Ti_3_C_2_T_x_ (T = -O,-OH or -F) after LiF/HCl etching of Ti_3_AlC_2_ and centrifuging for different times, with permission from Reference [24], copyright (2019) American Chemical Society. (**c**) The synthesis mechanism of Ti_3_C_2_T_x_ MXenes/nanocarbon-sphere hybrids. First, the Al layer is removed from the corresponding MAX phase by HF. Second, part of the carbon atoms are migrated to the surface of the Ti_3_C_2_T_x_ MXenes. This process can be controlled by using different HF treatment times, and microwave absorption effects are also exhibited. Used with permission from Reference [3], copyright (2018) The Royal Society of Chemistry.

Two-dimensional layered titanium carbide synthesized by different processes may exhibit different structure or morphology to further enhance or change their functional properties [3,5,24,73]. Another previously unrecognized new 2D tetragonal titanium carbide material (named tetr-TiC) with intrinsic metal properties has been studied by Fan recently. First-principle calculations have also been carried out according to this tetr-TiC structure, and the atomics configuration was presented as Figure 8a. The tetr-TiC is mechanically and dynamically stable, which thus suggests that tetr-TiC can be synthesized by the laboratory. More importantly, in addition to high stabilities and metallic properties, this new 2D tetr-TiC has a lower Li diffusion barrier (fast Li^+^ diffusion rate), higher theoretical capacity and lower average open circuit voltage compared with previously reported 2D Ti_2_C and Ti_3_C_2_ nanosheets [5]. He et al. successfully synthesized delaminated titanium carbide MXene nanosheet (named as D-Ti_3_C_2_T_x_ in this case) via a modified Gogotsi’s method. As presented in Figure 8b, they first used LiF/HCl as etching solution and the Ti_3_AlC_2_ was transform into multilayered Ti_3_C_2_T_x_ (named as M-Ti_3_C_2_T_x_ and T = -O,-OH or -F). Then, upon further increasing the centrifuge time, the layer distance also increased, and the M-Ti_3_C_2_T_x_ finally transformed into D-Ti_3_C_2_T_x_ [24]. The resulting atomic layers processing showed a special function—the tailoring of the Ti_3_C_2_T_x_ layer distance can significantly enhance the effect of polarization. Because of the conduction loss and polarization loss are competition processes, with increasing concentrations of D-Ti_3_C_2_T_x_ in the composites, the conversion rate of electromagnetic energy to thermal energy will also increase.

However, it is known that the conductivity of Ti_3_C_2_T_x_ MXenes is very high, which is not favorable to the demands of impedance matching and leads to strong reflection and weak absorption. Dai et al. designed novel 2D Ti_3_C_2_T_x_ MXenes structures to enhance the microwave absorption properties. The new 2D laminated structures are mainly Ti_3_C_2_T_x_ MXenes/nanocarbon-sphere hybrid structures, as shown in Figure 8c. The appearance of a nanocarbon sphere was mainly accounted for by the increasing time of HF treatment. In addition, these hybrids structures show excellent microwave absorption properties due to the heterogeneous interface structures [3].

In summary, the excellent microwave absorption, EMI shielding, energy conversion, catalysis, etc., properties and unique physical and chemical properties make these two-dimensional titanium carbides have immense potential for use in a multitude of areas. However, the study of MXene materials is novel work and still has some room for development. Therefore, examining the specific morphological transformations of 2D nanosheets of titanium carbides can be seen as a new tendency.

Although 2D titanium carbides seem to exhibit special crystal structures and morphologies, they are also closely related to the synthesis processes of 3D titanium carbide particles, which can be seen in the thermodynamic calculation of Al-Ti-C system [74,75]. For instance, the first step of the reaction with Al, Ti and C with specific ratios and synthetic parameters will form Ti_n+1_AlC_n_ (*n* = 2 or 3), which are the precursor phases (named MAX) mentioned previously. By using the HF to etch the Al layer in the Ti_n+1_AlC_n_ structure, the Ti_n+1_AlC_n_ phase may be transformed into MXenes (Ti_n+1_C_n_). However, in these MAX phases, when the original reactant ratio or some reaction thermodynamics and dynamic conditions are satisfied to form face-center cubic (FCC)-structured TiC*_x_*, some of the Ti_n+1_AlC_n_ phase may transform into FCC TiC*_x_*, which can be seen as a particle with a three-dimensional morphology [74].

## 5. The Synthesis and Characterization of Three-Dimensional Titanium Carbides as Well as Their Applications

Classified as three-dimensional nanomaterials, which means that the external size may be restricted by more complex nanoscales, titanium carbide nanoparticles can be well-defined in this case. Compared with the one or two-dimensional nanostructures with more complex morphologies synthesized in recent years, three-dimensional titanium carbide nanoparticles were studied much earlier during the past several decades. There are also abundant methods to synthesize the 3D nanocrystals, for instance, the chemical synthesis reaction, which is similar to the process used for 1D and 2D structure, or synthesis reactions in binary or ternary Ti-C-(Me) system (Me represent alloying elements). The TiC nanocrystal obtained by the reaction in Ti-C-(Me) system can be utilized as structural materials, especially for light-weight manufacturing including alloys refining and reinforcing. However, few articles have summarized the growth and morphological evolution of 3D TiC nanoparticles. Therefore, in this chapter, the sizes and morphologies of titanium carbide nanoparticles fabricated in Ti-C (mainly containing titaniferous compounds or carbides) [27,42] or Me-Ti-C systems (where ‘Me’ represents Al, Cu, Fe, Si and Ni) are summarized [13,16,17,18,76], and their applications in various fields are also discussed. Moreover, Ti and C are not limited to Ti or C powders, as various titanium and carbon sources are available during different chemical synthesis processes.

### 5.1. The Synthesis of Three-Dimensional Titanium Carbides in Me-Ti-C Systems (‘Me’ Represents Alloying Elements Al, Cu, Fe, Si and Ni)

In the conventional ceramic synthesis processes, reaction between Ti and C cannot occur unless an elevated temperature (higher than 3000 K) or some special synthesis route is used [77,78,79,80]. Such high ignition temperatures approaching the melting point of titanium carbide is hard to obtain and will cause a waste of energy. Choi and Rhee [77] reported that without the addition of Al, the morphology of the TiC*_x_* in Ti-C reaction system (molar ratio C/Ti = 1) was sintered-like and was not characterized by a typical dispersed particle morphology. As the Al content increased from 0 to 40 wt.%, the combustion synthesis temperature decreased, and the morphology of the TiC*_x_* changed from sintered-like to monodispersed sphere-like, while the size of the TiC particles decreased with increasing Al contents. Therefore, it can be seen that the addition of Al can not only reduce the combustion temperature but also act as a disperser of the particles. Moreover, the Al incorporated into the Ti-C system will act as a reactant, participating in the formation of TiC. Many researchers have found that the addition of a second component Me (Me = Al, Mg, Cu, Fe, Ni, Si, etc.) will effectively reduce the titanium carbide synthesis temperature due to the formation of Me-Ti-C ternary phases which have low melting points. Obviously, under the influence of second components, the growth sizes and morphologies of titanium carbides will become inherently variable.

#### 5.1.1. The Growth Behaviors of Titanium Carbides in the Al-Ti-C System

**Figure 9:**
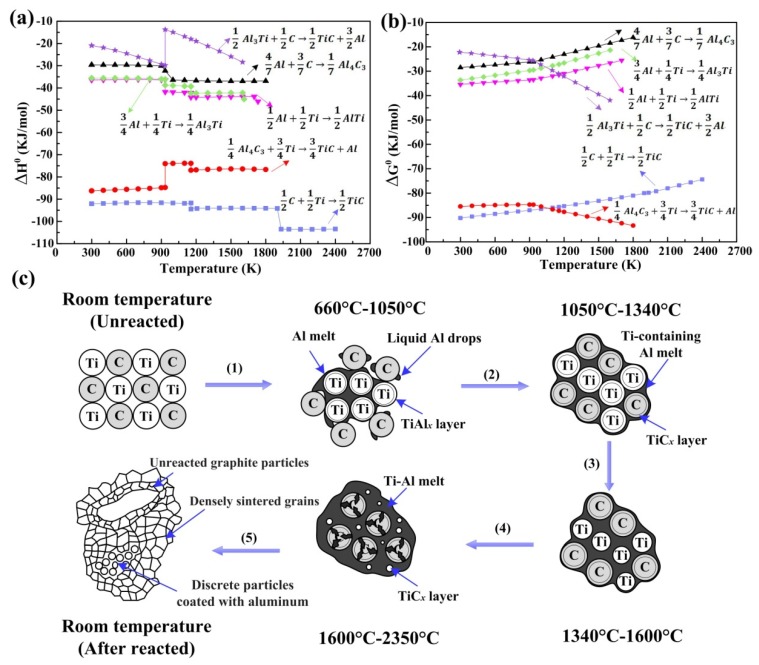
Changes in (**a**) reaction enthalpy, ∆H and (**b**) Gibbs free energy, ∆G in Ti-Al-C system; (**c**) Proposed mechanism of ignition and reaction of the Ti-Al-C system according to Lee et al.

The Al-Ti-C system is a classical reactive system and has been widely studied. Reactions in Al-Ti-C systems and their corresponding reaction enthalpies (∆H) and Gibbs free energies (∆G) are shown in Figure 9a,b, respectively. It can be seen that the formation of TiC releases more heat and shows the largest thermodynamic driving force. Therefore, this illustrates that TiC is a stable product even under elevated temperatures. As mentioned above, Choi et al. suggested that Al in the Al-Ti-C system can act as a reactant, and the reaction mechanisms can be summarized as follows:

(a) Titanium aluminide formation: 2Ti+ 2C + *x*A1 → TiA1*_x_* + Ti + 2C + *Q*_1_
(b) Titanium carbide formation: TiAl*_x_* + Ti + 2C → TiAl*_x_* + TiC + C + *Q*_2_
(c) Titanium aluminide decomposition: TiAl*_x_* + TiC + C → TiC + Ti + xA1 + C − *Q*_3_
(d) Titanium carbide formation: TiC + Ti + *x*A1 → C + 2TiC + *x*A1 + *Q*_4_

Lee et al. investigated the ignition phenomena as well as the reaction mechanisms in the Al-Ti-C system during the self-propagating high-temperature synthesis (SHS) process [78,80]. As Figure 9c illustrates, the reaction mechanism is summarized as follows. First TiAl*_x_* compounds form at the interface between the Al melt and Ti particles. Then, the Ti-containing Al melt spreads over the graphite, and a small number of TiC*_x_* layers form. As the temperature increases beyond the decomposition temperature of TiAl*_x_*, the TiAl*_x_* layers further decompose, and more Ti particles dissolve into the Al melt. After the complete melting of titanium, a Ti-Al liquid solution forms and infiltrates into the pores of the graphite particles. Therefore, the carbon atoms can diffuse through the TiC*_x_* layers to react with titanium, and more TiC phase will nucleate in the Ti-Al melt. However, there were some unreacted graphite particles in the final product, as shown in Figure 9c. Recently, Liu et al. found that ultrasound is able to promote combustion synthesis reactions in the Al-Ti-C system to be more complete [81]. High-intensity ultrasound could accelerate the formation of a saturated solution of C atoms in the Al–Ti melt, and the nucleation and growth of TiC could also be promoted. In addition, after ultrasound assistance, the amount of residual unreacted graphite particles decreased.

Similar to the study of Choi, Jiang et al., [14] also confirmed the analogous dissolution-precipitation mechanism in the combustion synthesis reaction. Many studies have illustrated that combustion synthesis in the Al-Ti-C system mainly depends on two processes. One occurs after the formation of the Al-Ti melt and involves the dissolution of the carbon source into the Al-Ti melt, while the other is the mass transfer rate in the Al-Ti-C melt during the nucleation and growth of the TiC phase. Both of these processes will finally influence the nucleation and growth of titanium carbides and then change their sizes and morphologies. In general, the investigations of reaction mechanisms mentioned above during synthesis are favorable for exploring the nucleation and growth behaviors of titanium carbides. Finally, the sizes and morphologies of titanium carbide will be well regulated and controlled.

**Figure 10:**
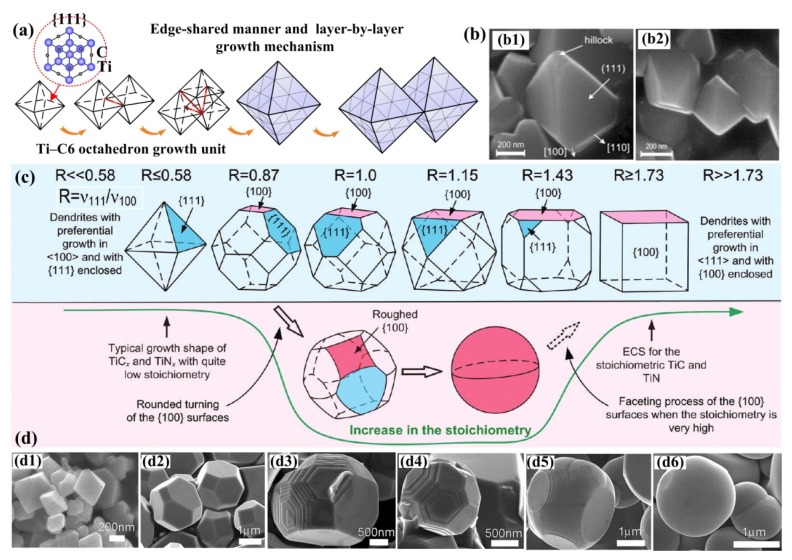
(**a**) A packing scheme of octahedral TiC in the edge-shared manner and layer-by-layer mechanism. (**b**) SEM images of TiC particles synthesized via SHS: (**b1**) a high magnification and (**b2**) an edge-sharing of octahedral TiC, with permission from Reference [14], copyright (2009) Elsevier; (**c**) Schematic illustration of TiC*_x_* growth morphologies. With permission from Reference [86], copyright (2012) American Chemical Society; (**d**) FESEM (Field emission scanning electron microscope) images of TiC*_x_* particles, with permission from Reference [13], copyright (2012) American Chemical Society.

Song et al. provided the formation and growth mode of TiC octahedrons during the self-propagating reaction, which can be concluded as edge-shared growth and layer-by-layer growth mechanisms, as shown in Figure 10a [82]. In their study, due to the TiC*_x_* nuclei deficit in carbon in the earlier step of nuclei growth, the TiC*_x_* growth driving forces at the corners or the edges were higher than those at the centers of the (111) facets, so the growth of corners or the edges was promoted, and TiC*_x_* formed a perfect octahedron. As Figure 10a shows, through edge-shared growth, more Ti-C_6_ octahedron growth units will enter the (111) facets of the intrinsic octahedron and link by the edges. Thus, the TiC*_x_* phases could repeatedly grow further. This linking process is similar to two-dimensional (2D) nucleation—the growth unit which enters into the (111) facet will generate a new step on the (111) facet, and other growth units entering into this (111) facet will cling to this newly formed step to grow sequentially. Because there exist some deficits in carbon at the corners or edges, some growth units have difficultly freely entering into the entire (111) facets. Therefore, a few hillocks or steps form on account of the growth units packing at the corners or edges, as observed in Figure 10b. They also suggested a vector relationship and a ratio R, which is the growth rate ratio of [100] to [111] (V_[100]_ to V_[111]_). It was found that when R = V_[100]_/V_[111]_ = 1.5, perfect octahedron TiC particles will be obtained. When R > 1.5, TiC particles are imperfect octahedrons. While R < 1.5, TiC particles with irregular shapes will be observed.

It was reported by Cochepin et al. [83] that the nucleation and growth of TiC*_x_* phases could take place under a great deficit of carbon. Additionally, in the study by Merzhanov [84], the initial state of combustion synthesis can be seen as a nonequilibrium process, and as the reaction proceeded further (after the maximum combustion temperature), the reaction tended to reach equilibrium. In general, under a great deficit of carbon, the TiC*_x_* nucleates had low *x* values, and the composition of carbon evolved from substoichiometric towards stoichiometric during the growth of the TiC*_x_* phase. Therefore, it can be predicted that this carbon dissolution and diffusion process may strongly influence the growth of the TiC*_x_* phase at all stages of reactions, which influences the stoichiometric ratios and, correspondingly, the morphologies of TiC*_x_*.

Jin et al. found a temperature-related growth behavior in the Al-Ti-C reaction system during the self-propagating high-temperature synthesis process, and they demonstrated that the TiC*_x_* particles transformed from octahedrons and truncated octahedrons to spheres upon increasing the temperature [13]. Their later studies illustrated that the intrinsic morphology evolution of TiC crystal was caused by the induction of stoichiometric ratio [30,85,86]. The corresponding evolutions are shown in Figure 10d. The morphology evolution induced by stoichiometric ratios, transforming from octahedral to truncated-octahedral, sphere-like and, finally, spherical can also be realized by increasing the carbon source content, which can be considered as the stoichiometric ratio. No cubic TiC*_x_* was observed due to the roughening transitions of (100) planes. By observing the growth behaviors during the growth of TiC*_x_*, Jin et al. summarized two growth modes according to different concentrations of carbon. (1) One occurs under a low carbon concentration, in which the Ti-C_6_ octahedron growth units laterally stack on the (111) crystal planes, which is analogous to the findings in the study by Song. However, this growth mode may affect by the formation rate of the new growth steps on the lateral growth layers. If the formation rate is too fast, the growth units on the former layer have no time to grow and pervade the (111) crystal planes, while later growth layers will form, so a truncated-octahedral will be obtained. (2) The other model is 2D nucleation growth on the (111) crystal planes, which can only be launched under a high carbon concentration, and the TiC*_x_* grows into a high stoichiometric ratio (C/Ti). The stoichiometric ratio increase will lead to more rapid growth in the TiC_x_ [111] crystalline direction than in the [100] crystalline direction. Hence, the result showed a morphological variation process which can be simplified as the shrinkage of the (111) crystal planes, as well as the exposure of the (100) crystal planes. In addition, a critical size of 2D nuclei should be attained in this case to surmount the energy increase when creating the edges. Additionally, a high carbon concentration can provide a high probability of C atom deposition and congregation on certain surfaces to reach the critical 2D nuclei size. Jin et al. distinguished these two growth models in detail according to the concentration of C, which has a great reference value to the crystal growth behaviors during combustion synthesis.

In addition to the octahedral or spherical shapes mentioned above, a kind of hexagonal platelet and macroporous morphology TiC*_x_* was reported in the Al-5Ti-0.3C master alloy. By using Al-5C alloy as the carbon source, via the solid-liquid reaction between the Al_4_C_3_ and the dissolved Ti, the synthesized TiC particles exhibited hexagonal platelet and macroporous morphologies, which were distributed homogeneously throughout the Al matrix [87]. The macroporous morphological evolution can be explained by the further dissolution of Al_4_C_3_, and through this growth mechanism, different hexagonal platelets will be observed. Additionally, compared with the conventional Al-5Ti-0.3C master alloy, their new Al-5Ti-0.3C master alloy showed a much better grain refinement effects on commercially pure Al. Moreover, within 30 min, the preferable refining efficiency of their new master alloy did not fade obviously.

Therefore, it can be seen that the carbon concentration plays a significant role during the reactions. Moreover, it is known that different carbon sources have different decomposition and diffusion activities, which will also become a controlling factor during the fabrication process. Graphite is a conventional carbon source and has a broad variety of sources. It isinexpensive and has good stability, while its average size is relatively large. In previous studies, graphite particles of approximately 5 μm, 15 μm, 38 μm or even 48 μm were reported as used to fabricate TiC [30,75,80,86]. Becasuse graphite has a large size, it always becomes a residual phase in the reactant and has not been completely reacted with Ti, except for via some special measures [81]. Wang et al. suggested that the residual graphite in the reactant was actually graphite flake agglomerations [87]. Carbon black is also a kind of a low-cost carbon source and has a smaller size than most graphite, which is approximately 0.1 μm. However, the dissolution rate of carbon black during the synthesis reaction is also low, which will influence the deposition process and is not conducive to the formation of TiC. Carbon nanotubes (CNTs) have been aroused wide consideration recently due to their specific properties, for instance, their relatively small sizes (such as diameters of 10 nm to 20 nm, and lengths of 20 nm to 100 μm) and large specific surface areas. Additionally, some structural defects such as vacancies, dopants, pentagons and heptagons exist in CNTs structures, which will give CNTs relatively high chemical activity. Some studies also suggested that the wettability between the metal melt and CNTs can be improved by the defects, which is more favorable for the dissolution and diffusion of carbon sources in the melt, and then reactions between Ti and the CNTs can be easily ignited and conducted [88,89,90,91].

**Figure 11:**
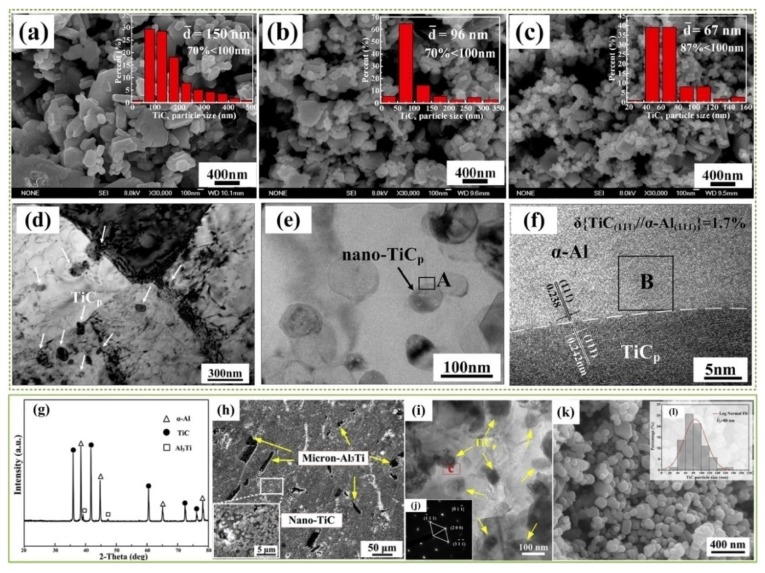
The nanosized TiC*_p_* extracted from the as-synthesized TiC*_p_*/Al-Cu-Mg-Si nanocomposites produced using different carbon sources: (**a**) carbon black, (**b**) mixed carbon source (50 wt.% carbon nanotubes (CNTs) + 50 wt.% carbon black), and (**c**) CNTs. The TEM images of the 10 vol.% TiC*_p_*/Al-Cu-Mg-Si nanocomposite (fabricated by mixed carbon source) after tensile testing at 298 K: (**d**) the distribution of TiC*_p_*, (**e**) the morphology and interface of the observed TiC*_p_*, and (**f**) the corresponding TiC*_p_*/α-Al interface bonding. With permission from Reference [92], copyright (2019) Elsevier; (**g**) The XRD phase analysis and SEM micrographs of (**h**) the Al-30 vol.% (TiC_n_-Al_3_Ti_m_) master alloy, (**i**) the TEM image of nano-TiC particles in the master alloy, (**j**) nano-TiC particle selected area electron diffraction (SAED) pattern, (**k**) A FESEM micrograph of the extracted TiC particles morphology and (**f**) TiC particles size distribution statistical histogram; With permission from Reference [19], copyright (2019) Elsevier.

Due to the distinctions between the carbon sources, has been found that the sizes of as-synthesized TiC particles are strongly dependent on the initial size and activity of the carbon sources. Gao et al. found that the as-synthesized TiC particle sizes decreased gradually as the carbon sources was changed from pure carbon black to mixed source (50 wt.% CNTs + 50wt.% carbon black) and then to pure CNTs. Taking 10 vol.%TiC/Al-Cu-Mg-Si as a example, the size of TiC nanoparticles formed with carbon black was 150 nm on average, and that formed with a mixed source was about 96 nm, while that using CNTs was about 67 nm. As shown in Figure 11a–c, TiC nanoparticles synthesized using different carbon sources and 10 vol.%TiC/Al-Cu-Mg-Si were extracted and observed by FESEM. The results suggest that during the ball milling process, CNTs will form bundles and tangles, which result in a non-uniform distribution of [C]-rich regions. When carbon black was used as the carbon source, it was demonstrated that carbon black, with its lower decomposition and dissolution rates, was also not favorable for the uniform distribution of [C]-rich regions. Therefore, the mixed carbon source could endow the most homogeneous distribution of the precipitated nano-TiC*_p_* after combustion synthesis in the Al-Ti-C system. Figure 11d shows that the TiC*_p_* (fabricated by mixed carbon sources in 10 vol.%TiC_p_/Al-Cu-Mg-Si through hot extrusion at 833 K and T6 heat treatment) will distribute in the α-Al grains or at the grain boundaries after tensile testing. Good interface bonding could be observed between TiC*_p_* particle and Al matrix, as Figure 11e,f demonstrate, the mismatching between Al(111) and TiC*_p_*(111) was only 1.7%. Also, the TiC*_p_*/Al-Cu-Mg-Si nanocomposites fabricated using the mixed carbon source exhibited the best mechanical properties [92].

A similar fabrication and application of TiC nanoparticles was reported by Li and Tian et al. [19,29] Without a further hot extrusion process, the as-synthesized TiC*_p_* is directly incorporated into Al and its alloys. A kind of master alloy has been fabricated by Li et al. by combustion synthesis, which included 30 vol.% (nano-TiC + micron-Al_3_Ti) and 70 vol.% Al. The XRD phase analysis and the corresponding microstructure of the master alloy are shown in Figure 11g–h. The TiC nanoparticles were distributed uniformly in the Al matrix, as Figure 11i suggests, and the SAED analysis demonstrated that the uniformly distributed particles were TiC. The extracted TiC nanoparticles in Figure 11k illustrated that the nano-TiC fabricated in this case showed mainly spherical morphologies with the size of 80 nm on average. Li et al. found that those nanosized spherical TiC particles can exert a significant role in inhibiting the growth of α-Al dendrites, and therefore, grain refinement could be realized. Under their observations, the nano-TiC were pushed by the solid-liquid interface and distributed along the α-Al grain boundary. As a result, the low lattice mismatching between the α-Al crystal surface and the exposed surfaces of the TiC particles suggests a good wettability of the TiC particles in the aluminum melt, so more TiC*_x_* ceramic particles will act as heterogeneous nucleation sites, promoting α-Al heterogeneous nucleation. Other TiC particles which do not act as nuclei can effectively impede the solute diffusion, finally restricting the further growth of α-Al grains. Analogously, grain refinement has been extensively reported as realized using TiC particles [1,2,29,92,93,94]. In the study by Tian et al., bimodal-sized micron-TiC and nano-TiC particles exhibited excellent refinement and strength abilities. The creep resistance of Al-Cu alloy has also been improved by the addition of TiC*_p_* [29]. The addition of nanosized TiC particles can effectively refine the *θ*′ precipitates (Al_2_Cu) of Al-Cu, which was also favorable for improving the properties of the Al-Cu alloy [93]. Yang et al. observed that spherical nanosized TiC may hinder the transformation of dislocations, enhancing the strength of the alloy to some extent [2]. Those nanosized 1.5%TiC particles could also effectively improve the age-hardening of Al–4.5Cu, and the peak-age times of Al–4.5Cu–1.5TiC were also decreased [95].

To accurately determine the relative stabilities of the (100) and (111) planes, some first-principle simulation works have been performed from the perspectives of atoms, surfaces and interfaces [96,97,98]. Based on different TiC*_x_* stoichiometric ratio (*x* = 0.5, 0.625, 0.75, 0.875, 1.0), Zhou et al. successfully calculated the surface energies of the (100)_TiC*x*_ and (111)_TiC*x*_ surfaces by the density functional theory, assisted by the generalized gradient approximation (GGA) method [97]. It was found that with the increase of the TiC*_x_* stoichiometric ratio, the surface energy of the (100)_TiC*x*_ decreased more quickly than did that of the (111)_TiC*x*_, and the (100)_TiC*x*_ surfaces tended to be more stable under high stoichiometric ratios. Therefore, it can be predicted that (100)_TiC*x*_ surfaces will gradually be exposed while (111)_TiC*x*_ surfaces will gradually shrink and disappear. The morphology evolutions are based on the relative stabilities of different crystal planes.

In fact, the relative stability of the dominating low-index crystal planes in FCC titanium carbide is relative. Mao et al. used the first-principle method to calculate the surface energy of TiC, which considered the surface orientation, surface termination, chemical potential of carbon, influence of various concentrations surface vacancy defects, and electronic structures. They found that for surfaces without defects, the order of the low-index surface stabilities depended on the carbon chemical potential. Under low carbon chemical potential, the order of stability was C-terminated (111) < (110) < (001) < Ti-terminated. However, when the chemical potential of carbon was high, the stability orders was C-terminated (111) < (110) < Ti-terminated < (001). For surfaces with defects, the stability order was the same under different carbon chemical potentials, following C-terminated (111) < Ti-terminated (111) < (110) < (001). It can be seen that those analytical results are favorable for the control of the nanoparticle morphology and for promoting the catalysis performance of titanium carbide by carefully changing the carbon sources during synthesis [35].

**Figure 12:**
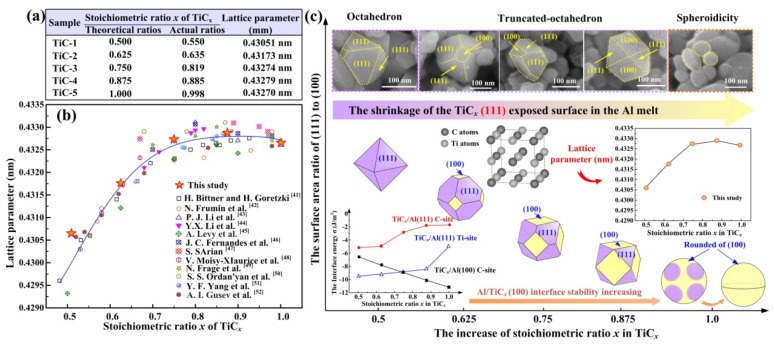
(**a**) The theoretical and actual stoichiometric ratios and the corresponding extracted TiC*_x_* lattice parameters; (**b**) The lattice parameters and corresponding estimated stoichiometric ratios of TiC*_x_* in this case compared with previously reported experimentally determined data; (**c**) The TiC*_x_* nanoparticles morphology evolution manipulating mechanism by the stoichiometric ratios in the Al melt. With permission from Reference [101], copyright (2019) Elsevier.

In summary, whether by changing the carbon sources or controlling the different addition contents of carbon in the reaction systems, the goal is regulating the constituents and diffusion of C in the melt to realize the deposition of the Ti-C phase. In this way, TiC*_x_* materials with different stoichiometric ratios are accessible and their morphologies will be varied during the reactions. The corresponding chemical and physical properties of TiC*_x_* will also provide some differences in their applications. Vasanthakumar et al. found that the lattice parameters, as well as the intensity ratios of the {200}_TiC*x*_ to {111}_TiC*x*_ peaks in XRD analysis, were related to the ratio of C to Ti. As the ratio of C/Ti increased, the elastic modulus and hardness were also enhanced [98]. Yang et al. investigated the relationship of C/Ti stoichiometric ratios, (which means the value of *x* in TiC*_x_*) and grain refinement efficiency using five Al-5Ti-*m*C master alloys. Here, *m* represents 0.1, 0.5, 0.8, 1 and 1.25, respectively. It was suggested that the Al-5Ti-*m*C master alloy with a lower *x* in TiC*_x_* exhibited a better refinement efficiency and anti-fading capability due to the formation of Ti-rich zone around the lower *x* TiC*_x_* melt interface when incorporated into the melt [99]. Qiu et al. fabricated 50 vol.% TiC*_x_*/2014Al in the Al-Ti-C system where the reaction ratios of C to Ti were 0.6 to 1.0. It was found that when the molar ratio of C/Ti was 0.8, the composites showed the best wear resistance and compression properties [100]. In our previous work, TiC*_x_* nanoparticles were fabricated with desirable morphologies in Al melt by combustion synthesis in the Al-Ti-C system. The corresponding morphology manipulating mechanisms of TiC*_x_* nanoparticles by stoichiometric ratio *x* (*x* = 0.5, 0.625, 0.75, 0.875, 1.0) were revealed by experiment and first-principles calculation. On one hand, it can be seen that the actual stoichiometric ratios of the extracted TiC*_x_* in Figure 12a may differ from the theoretical reactant ratios. The measured lattices parameters compared with previously reported experimentally determined data are shown in Figure 12b, which shows the actual stoichiometric ratios. On the other hand, in this case, the Al/TiC*_x_* interfaces were the main focus, and a designed growth morphology manipulating mechanism of TiC*_x_* nanoparticles was presented. As *x* increased from 0.5 to 1.0, the interface energy of the Al/TiC*_x_* (100) C-site decreased gradually, and the electronic hybridization between Ti*_3d_* and C*_2p_* became stronger. In addition, the Al-C or Al-Ti bonding in the (111) crystal planes of the Al/TiC*_x_* interface showed better strength when *x* was 0.5 rather than 1.0, which suggests that the (111) bonding interfaces are dominant under low stoichiometric ratios. Therefore, as the stoichiometric ratio increased, the (100) crystal planes of TiC*_x_* gradually stabilized and exposed while (111)_TiC*x*_ shrank and disappeared in the Al melt. The corresponding morphologies of TiC*_x_* evolved from octahedrons, and truncated-octahedrons to spheroids in Al melt with the increase of *x* from 0.5 to 1.0, as shown in Figure 12c. By manipulating the specific stoichiometric ratios *x*, the growth behavior of TiC*_x_* nanoparticles can be artificially intervened and controlled, which has realistic significance. More importantly, through manipulation of the synthesis processes, TiC*_x_* nanoparticles can be preferentially synthesized with desirable morphologies and specific exposed surfaces to meet their broad development and application prospects [101].

Moreover, the dissolution-precipitation process is a nonequilibrium process, which is accompanied by changes of the stoichiometric ratio. Obviously, the nonequilibrium characteristics shown by the TiC*_x_* during combustion synthesis processing, such as the significant temperature gradient, extremely high reaction temperature, extremely fast reaction speed, nonlinear and unsteady transfer processes (including energy, mass and momentum transfer) are responsible. Moreover, in addition to SHS, there are various synthesis methods by which to fabricate TiC*_x_*. However, the synthesis processes are always followed by complicated physical and chemical processes, and the growth morphology of TiC will be influenced significantly by many factors, such as the reactants categories and contents, reaction temperatures and atom diffusion rates mentioned above. Therefore, it can be seen that the addition of alloying elements can also significantly change the melting environment.

#### 5.1.2. Other Influencing Factors that Change the Morphology of Titanium Carbide in the Al-Ti-C System

**Figure 13:**
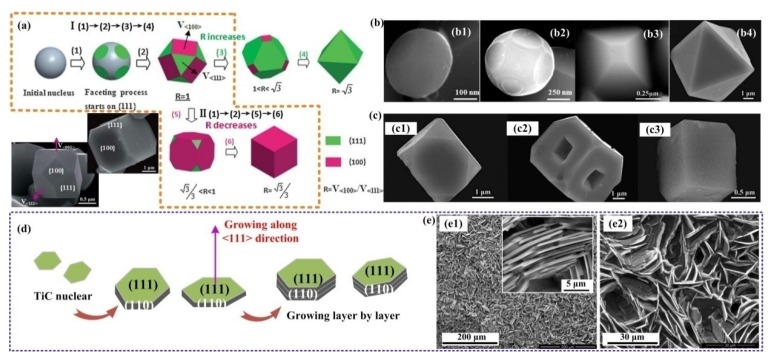
(**a**) TiC morphology evolution model schematic illustration in Al-Ti-C (Route I) and Al-Ni-Ti-C systems (Route II). (100) and (111) faces are shown in green and purple, respectively. (**b**) The possible morphology of TiC*_x_* in the Al-Ti-C melt. (**c**) The morphology of TiC*_x_* under the influence of Ni, with permission from Reference [15], copyright (2012) Royal Society of Chemistry. (**d**) Hexagonal TiC platelet growth schematic illustration. (**e**) TiC platelets (**e1**) formed on the surface of the Ti/Si/TiC/Al_0.2_ sample (obtained after 1500 °C sintering for 10 min in Ar atmosphere) and (**e2**) on the surface of Ti/Si/Al_0.2_ (obtained after 1450 °C sintering for 10 min), with permission from Reference [16], copyright (2008) Elsevier.

Some additional elements in the melt can bring significant effects to the growth behavior of titanium carbide. Nie et al. [15] found that the TiC particles morphologies produced in Al-Ti-C and Al-Ti-Ni-C systems are totally different. In the Al-Ti-C system, the growth process of TiC, which can be simplified as route I in Figure 13a, will gradually transform the product from a near-sphere (as Figure 13(b1,b2) suggested) to the perfect octahedron and truncated-octahedron in Figure 13(b3), and the octahedron in Figure 13(b4) is produced by this transformation. Upon adding Ni into the Al-Ti-C system, the growth process follows route II in Figure 13a, with the product finally evolving into a cube. Since there are strong interactions between Ni-3d and C-2p orbitals, the added Ni tends to absorb on the (100)_TiC*x*_ planes rather than the (111)_TiC*x*_ planes, so the growth rate of (111) is accelerated while the growth of (100)_TiC*x*_ is restricted. In this case, the total surface free energy of (100)_TiC*x*_ is the smallest. Through the intermediate evolution process (as Figure 13(c1) exhibits), the TiC*_x_* particles presented as cubes enclosed by six (100)_TiC*x*_ planes. The presence of some cubic TiC with hoppers may be because the TiC particles in the Al-Ni-Ti-C alloy have a faster growth rate in (111) than in (100). Similar to the conclusion of Song et al., an octahedron growing unit takes on a significant role during growth, while the addition of Ni will break the equilibrium growth process. The growth rate on the eight edges of each growing unit was faster than that on the (100) planes, so symmetrical hollows will appear on the face centers of the cubic skeleton during the growth process of TiC, as Figure 13(c2) suggests. With further growth, the symmetric hollows will shrink and disappear, and a perfect TiC cubic as shown Figure 13(c3) will be obtained. No matter how complex the reactions are, the final morphology of the TiC particles is mainly related to the ratio of the growing rate of each facet or edge.

Nie et al. also demonstrated a similar selective adsorption effect of the group VIII transition-metal elements Fe and Co when doped into the Al-Ti-C melt to form TiC. Notably, the doping impurity atoms can absorb on a specific plane and even cover the whole surface, and the surface energy is also changed. Therefore, moving these impurity atoms away from the crystal planes will require more energy, which finally retards the growth rate. This research also gives us a comprehensive and in-depth exhibition of how the alloying elements Ni, Fe and Co affect the growth of TiC particles. This selective adsorption effect of the impurity elements on the specific crystal planes will remarkably restrict the relative growth ratios of different crystal planes, which can give us a convenient way to control the crystal growth. [15]

Mg is known as a kind of alloying element with a relatively low melting point. Therefore, the addition of Mg into the Al-Ti-C reaction system will induce a low combustion temperature during the combustion synthesis. Wang et al. found that the addition of Mg into the Al-Ti-C system will weaken the full conversion of Ti and C to TiC. As the Mg content increased from 0 wt.% to 5 wt.%, the combustion temperature decreased, which caused the size of the TiC particles to decrease. They suggested that the addition of Mg may reduce the mass transfer function of Al in the Al-Ti-C-Mg system (Mg and Al will easily form the Mg_17_Al_12_ phase, so the content of Al which participates as reactant in the reactions will be reduced) and suppress the growth of TiC, finally causing finer-sized TiC [102].

Li et al. found that small amounts of silicon and aluminum can influence the structure of TiC by dissolving into the TiC crystal lattice [16]. In their study, after sintering above 1450 °C under an argon atmosphere, abundant hexagonal platelets TiC were formed on the surface of a Ti/Si/TiC/Al_0.2_ sample, as shown in Figure 13(e1), and a Ti/Si/Al_0.2_ sample, as Figure 13(e2). The addition of TiC was used to compare the existence of an additional carbon source, but interestingly, they found that the main carbon source was from graphite dies. The formation of the stacking hexagonal platelets morphology may have been due to the influence of Al and Si, which will induce high-density planar defects within the TiC crystal. Additionally, Ti and Si will form Ti-Si liquid when the temperature exceeded 1330 °C, and Ti-Ti_5_Si_3_ and Si-TiSi_2_ eutectic reactions both occur at 1333 °C, so hexagon-shaped TiC nuclei could grow in the liquid environment at all the times. After the hexagonal platelet TiC started to nucleate, Ti and C atoms arrived at the surface of the former surface further deposited and reacted to form the next TiC nucleus in a continuously cycle. Hence repeatedly, as illustrated in Figure 13d, the stacking hexagonal platelet TiC will grow together along the <110> direction and form thick hexagonal TiC platelets. Other work demonstrated that in comparison with silicon, aluminum is also favorable to reduce the TiC twin boundary energy. This may be because the π-bonding between the carbon neighbors with Al is stronger than that with Si [103].

Moreover, Si will destabilize TiC. When Si is added into the Al-Ti-C alloy, the decomposition process will be accelerated. However, Ding et al. found that Al_4_C_3_ can be easily formed in the Al–Ti–C–Si alloy by the destabilization of TiC, while the simultaneously formed TiAl_x_Si_y_ phase will poison the refining efficiency [94]. Zhang et al. used the pyrolysis of PSCC mixed with Ti and TiSi_2_ to fabricate TiC with different morphologies. Similar to the studies using Al-Ti-C, the morphology of TiC also directly resulted from the raw materials carbon composition. The crystalline TiC showed a morphology that gradually evolved from perfect octahedrons to truncated octahedrons, and hexagonal pallets as the C concentration in the raw materials increased. In addition, the diffusion of silicon atoms into the crystal lattice of TiC will make the diffraction peaks of TiC deviate from the standard diffraction peaks. Zhang et al. also found that the addition of CaF_2_ could make TiC with an octahedral morphology transform into a near-spherical morphology [76].

It is known that B, N, O and other atoms which have similar atomic radii to that of C can diffuse into the TiC crystal lattice and occupy C vacancies or substitute C sites. This phenomenon may occasionally bring a few poisoning problems, but more often, it is an opportunity to improve the characteristics of TiC*_x_*. Chien demonstrated that C, N and O mainly substitute vacant carbon sites, while B mainly forms borides that precipitate from the reaction system. However, this is not absolute, because some studies suggested that several B atoms can also diffuse into the TiC*_x_* [104].

Nie et al. demonstrated that B atoms which exhibit a similar atomic radius will diffuse into the TiC*_x_* crystal lattice and occupy the sites of carbon vacancies, and this B-doped TiC*_x_* can still maintain the face-centered cubic structure. This phenomenon can enhance the stability of the TiC*_x_* crystal lattice and make the (200) plane of TiC*_x_* the strongly preferential orientation, which could give this B-doped TiC*_x_* a better refining performance and effective refining holding time on commercially pure Al [105]. Therefore, it can be seen that a few B atoms can bring a significant change to TiC_x_. It is known that after the substitution of C vacancies by B atoms, B atoms will prevent C atoms from moving away from the TiC_x_ crystal lattice, so the decomposition of TiC*_x_* in the melt can be alleviated [94]. Further studies by Nie et al. suggest that the structural evolution from TiC*_x_* to TiB_2_ can be realized by a relatively large addition of B. The possible evolution mechanism can be seen as consisting of three steps, including (1) TiC*_x_* delamination; (2) TiB_2_ in situ crystallization on the TiC*_x_* nanolamellas; and (3) corresponding oriented attachment and Ostwald ripening during the TiB_2_ growth process [106].

Doping with N can also stabilize the crystal structure of TiC*_x_*. Zhang et al. prepared an Al-Ti-C master alloy with N doping. In this case, nitrogen from liquid air (i.e., without protection gas) was doped into the TiC*_x_* during the sintering process in a sintering furnace with a slow heating rate [107]. A slow heating rate was favorable for the diffusion of N into the TiC*_x_* crystal lattice. Using the high-frequency furnace, due to the fast heating, N atoms had fewer chances to diffuse into the TiC*_x_*. However, when using the same sintering furnace under the protection of N_2_, the excessive N reacted with Al to form AlN, which hindered the doping process of N into TiC*_x_*. As Chien [104] et al. suggested, the lattice distortions which were caused by carbon vacancies could alleviate by the doping of nitrogen atoms into the TiC*_x_* lattices, so the doping processes can renovate defects in the face-centered cubic crystal structure. Concerning the result of Zhang et al., both excessive and insufficient nitrogen concentrations are adverse for the doping of N. Their results also demonstrated that the doping of N into TiC*_x_* lattices will not change their original crystal structures, but the lattice parameters will decrease compared with those of nondoped TiC*_x_*. More importantly, the N-doped TiC*_x_* particles showed obviously enhanced refinement efficiency and stability compared to nondoped TiC*_x_*.

Even acid solution immersion treatment will influence the crystal structure and stoichiometric ratio of TiC*_x_*. Heidarpour et al. used hydrofluoric acid (HF) solution to immerse the TiC*_x_* particles prepared by Ti-Al-C mechanical alloying and investigated the shape evolution of the TiC*_x_*. They found that the initial spherical shape of TiC*_x_* changed to a truncated octahedron after immersion for 24 h in the HF solution. When the immersion time was increased to 96 h, the octahedral morphology TiC*_x_* transformed to a cubic shape, and some sheet-like or branched morphologies of TiC particles were visible. Immersion into HF solutions could break the C-Ti bonds via fluoride ions, so after a long immersion time, cubic particles with some layered structures will appear. It turns out that this transformation may result from the change of the stoichiometric ratio. The corresponding stabilities of the (100) and (111) planes are also changed with the variation of the stoichiometric ratios [108].

#### 5.1.3. Reactions in the Cu-Ti-C System to Synthesize Titanium Carbides with Different Sizes and Morphologies

Copper is another important engineering and functional material due to its high electrical and thermal conductivity, high ductility, better formability, inherent corrosion resistivity and low cost (compared with Au and Ag), and it has been widely used as an electrical and thermal conduction functional material. However, the intrinsic poor wear resistance and low strength and hardness will impede extensive applications of Cu alloys. In recent years, nanosized ceramic particle-reinforced Cu matrix composites have exhibited excellent high-temperature mechanical properties and maintained their electrical and thermal conductivity. Titanium carbides can also display their excellent characteristics in Cu-matrix composites to further improve their comprehensive properties, and these composites are widely used as electrical contacts, heat sink materials, lead resistance wires, welding electrodes, etc. [109,110,111,112]

It is known that the Cu-Ti-C system is also a significant system used to obtain TiC*_x_*. The synthesis mechanism in the Cu-Ti-C system during the self-propagating high-temperature synthesis (SHS) was investigated by Liang et al. This reaction can also be seen as a kind of dissolution-precipitation process. The Cu-Ti-C system SHS reaction starts with the Cu and Ti solid diffusion reaction, and then a Cu-Ti liquid will form and cover the C particles. Later, the C particles will dissolve into the Cu-Ti liquid and form a Cu-Ti-C ternary liquid. Finally, C is consumed completely, and TiC particles gradually precipitate from the Cu-Ti-C liquid [113].

Akhtar et al. fabricated copper matrix composites reinforced by high-volume titanium carbide. The titanium carbide particles were distributed uniformly in the Cu matrix phase, and these copper matrix composites can be good candidates for sliding contact areas [114]. However, due to the different melting environment, the growth of TiC in the Cu-Ti-C system is different from that in the Al-Ti-C system.

**Figure 14:**
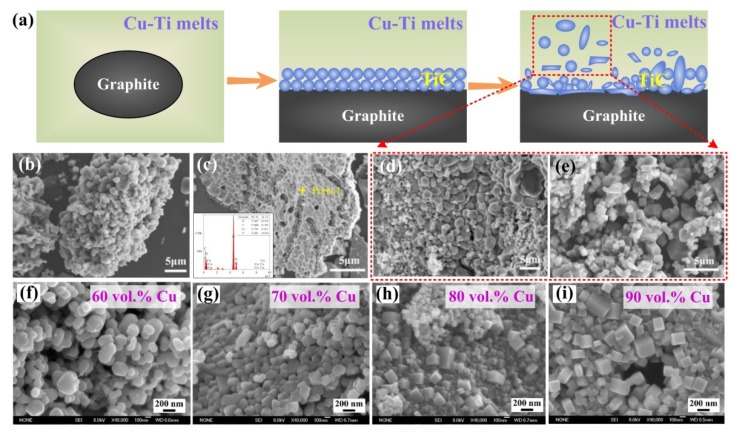
(**a**) the schematic diagram of the formation and separation of TiC in Cu-Ti melts. Some TiC that fails to break away from the graphite will form (**b**) TiC agglomerations or (**c**) partly integrate into larger plate-like morphologies. (**d**) Spherical and (**e**) polyhedron-like TiC particles in melts. With permission from Reference [115], copyright (2017) Elsevier.; (**f**–**i**) The extracted TiC particles synthesized by Cu-Ti-C system with different Cu content (from 60 vol.% to 90 vol.%).

Wang et al. synthesized TiC in a Cu-Ti melt by the reaction of graphite and soluble Ti during the melting-casting method [115]. They found that TiC mainly grew on the graphite, as Figure 14a–e suggests. Sine TiC particles separate from the graphite at different growth stages, TiC will take on different sizes and morphologies (spherical, as shown in Figure 14d, or polyhedron-like, as shown in Figure 14e), the schematic diagram of the formation and separation of TiC in Cu-Ti melts is exhibited in Figure 14a. Then, as presented in Figure 14b,c, some TiC particles which fail to break away from the graphite and drift into the melts will package with each other and combine into diverse agglomerates or integrate into larger plate-like shapes. Eremina et al. used graphite as the carbon source, and through the co-grinding of Ti and Cu powders and subsequent sintering, titanium carbide with cubic shapes, hexagonal titanium carbohydrides and Cu-Ti intermetallic phases could be synthesized [116].

The stability and transformation between two different stoichiometric ratios TiC*_x_* and TiC*_y_* in Cu–Si melts were investigated by Ding [117]. Here, they named the near-stoichiometric ratio TiC with fewer carbon vacancies as TiC*_x_*, which is the compound that is more stable in the Cu-Si melt. Nonstoichiometric TiC with many carbon vacancies was unstable and named TiC*_y_*. The reaction in Cu-Ti can be seen as:

TiC*_y_* → TiC*_x_* + Ti (*x* > *y*) (4)

When Ti in the Cu-Ti melt is excessive, the reaction will shift to the left, and TiC*_x_* tends to transform into TiC*_y_*. The addition of Si into the Cu-Ti-C melt will also influence the equilibrium of the reaction (4). This phenomenon may be due to the formation of CuTiSi or Ti_5_Si_3_ intermetallics, which consume much Ti and make the reaction shift to the right. Therefore, it can be seen that TiC with a large stoichiometric ratio shows better stability in the Cu-Si melt, and TiC with a large number of carbon vacancies will transform into TiC with few carbon vacancies. Their study provided a method to control the stoichiometric ratio of TiC. Qiang et al. also found a similar transformation of TiC in a Cu-Ti melt, in which excessive Ti is needed for near-stoichiometric (higher C/Ti) TiC*_y_* to transform into substoichiometric (higher Ti/C) TiC*_x_* [118].

Zhang et al. fabricated TiC*_x_* with different stoichiometric ratios (*x* = 0.4, 0.6, 0.8, 1.0 and 1.2) in a 70 vol.% Cu-Ti-C system via thermal explosion (TE) and hot press (HP) methods. In the samples with *x* < 0.6, the TiC*_x_* particles exhibited close-to-octahedral shapes, and when 0.6 < *x* < 1.0, close-to-spherical TiC could mostly be observed mostly. When *x* > 1.0 or Cu content > 80 vol.%, the cube-shaped TiC*_x_* particles could be obtained. They illustrated that carbon-rich areas can promote the formation of cube-shaped TiC*_x_* during in-situ synthesis. Therefore, under a high stoichiometric ratio or a high Cu content (a high Cu content will provide a low combustion temperature and a slow reaction speed, which gives the carbon source enough time to dissolve into the Cu-Ti melt), the growth of particles occurs in C-rich area, and TiC*_x_* can grow into a typical face-centered cubic crystal structure without or with a little carbon vacancies [26]. The morphologies of the TiC particles synthesized in Cu-Ti-C systems with different Cu contents (60–90 vol.%) are shown in Figure 14f–i, and the morphology evolution from close-to-spherical to cubic can be observed.

#### 5.1.4. The Reactions in Fe-Ti-C, Si-Ti-C and Ni-Ti-C Systems to Synthesize Titanium Carbides

**Figure 15:**
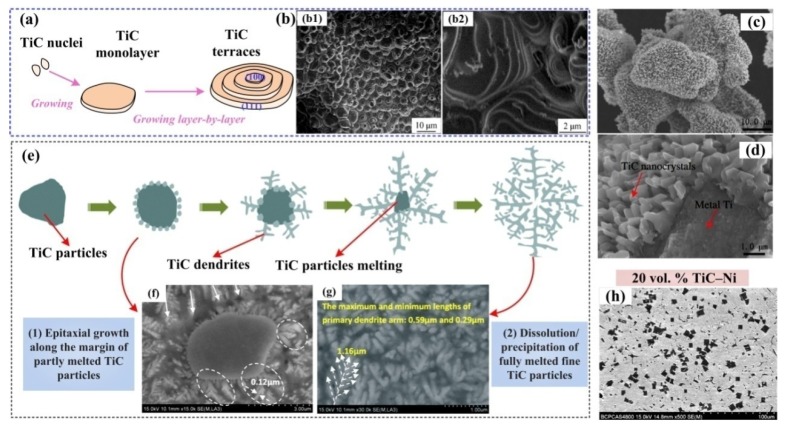
(**a**) Scheme of the growth process of TiC terraces in Fe-Ti-C system. (**b**) The morphology of TiC terraces synthesized in the Fe-Ti-C system. With permission from Reference [18], copyright (2011) Elsevier; (**c**,**d**) The TiC nanocrystal clusters that grow on the surface of Ti particles, with permission from Reference [120], copyright (2015) Elsevier; (**e**) Based on the TiC particles complete melting mechanism, the integrated dendrite development schematic diagram. (**f**,**g**) The actual TiC particles melting and dendrite development. With permission from Reference [17], copyright (2017) Royal Society of Chemistry; (**h**) The microstructure of 20 vol.%TiC/Ni composites. The large cubic TiC is the primary phase, while the fine fibrous shape is the eutectic phase. With permission from Reference [122], copyright (2010) Elsevier.

In addition to Al and Cu, Fe is another significant alloying matrix. Zhang et al. in situ synthesized TiC monolayers and terraces with 10 wt.% Fe-Ti-C elemental powder mixtures via a self-propagating high-temperature synthesis (SHS) reaction. First, the lattice parameter of TiC decreased because of the dissolution of Fe into the TiC lattice. Fe atoms in the TiC, lattice which are smaller than Ti atoms and larger than C atoms, will occupy the Ti sites rather than the C sites, so the Bragg angles will increase. They also revealed the formation and growth mechanism in Fe-Ti-C system to prepare TiC during the SHS process. As indicated, C first dissolved into the Fe–Ti melt and then TiC precipitated from the saturated melt. The thin TiC monolayer was grown by two-dimensional (2D) nucleation growth. Then the TiC terraces exhibited in Figure 15a,b show the TiC growing along the [100] direction through the layer-by-layer mechanism. Here, the growth mechanism of TiC is similar to that found by Jin et al. under a high C concentration in the system, in which TiC tends to grow by two-dimension (2D) nucleation growth and then grow layer by layer. As mentioned before, TiC may be form metastable TiC*_x_* nuclei preferentially when the content of carbon is in deficit, then the initial TiC*_x_* nuclei grow, and the TiC*_x_* grain evolves toward the stoichiometry along the reaction stage. During the TiC growth stage, the favorable temperature and time for the dissolution of C atoms into Fe-Ti liquid gradually decreases the creation of steps as well as the growth velocity of TiC (100) surface Therefore, the dimensions of the TiC monolayer decreases from the bottom to the top. They demonstrated the reaction mechanism in Fe-Ti-C, which can be summarized as follows (using the 30 wt.% Fe-Ti-C system as a case) [18]:

Fe + Ti + C → Fe + Fe_2_Ti + Ti + C → Fe + Fe_2_Ti + TiC + Ti + C → Fe + TiC (5)

This process can also be seen as a solution-precipitation mechanism under a high content of Fe. Similar to the Al-Ti-C system, in this case, with the increase of the Fe content from 10 wt.% to 40 wt.%, the as-synthesized TiC gradually transforms from layered terraces to near-spheres.

Lee et al. [119] used Fe alloy powder, TiH_2_ and carbon black powder to in-situ fabricate TiC via sintering at 1400 °C, and then further density tests of the sintered compacts were conducted using a the hot isostatic press (HIP).The in situ TiC showed good interface bonding with the Fe alloy matrix. Through the control of different C/Ti ratios in the reactants, the in situ TiC reinforced Fe alloy showed different hardnesses, flexural strengths and flexural strains. These variations may have come from the differences between C/Ti ratios and the actual stoichiometries, and either too much residual carbon or insufficient carbon during the synthesis was not favorable for enhancing the hardness and strength of the Fe alloys. However, they found that no matter the C/Ti ratio, in situ TiC particles were fabricated in the Fe alloy matrix with globular shapes and uniform dispersions.

In the study of Nie et al. mentioned previously, TiC particles fabricated in the Fe-Ti-C system will grow into perfect cubic morphologies. Additionally, in Al-Ti-C systems without alloying elements, TiC particles finally grow into octahedrons. However, in the Al-Fe-Ti-C system, TiC with a truncated cubic morphology can be obtained. Therefore, it can be seen that the competition between Al and Fe atoms can account for these truncated-cubic morphologies [15].

In the Ti-Si-C system, Zhang et al. fabricated TiC via the reactive pyrolysis of a mixture of PSCC, Ti, and TiSi_2_ particles under Ar atmosphere. The diffusion of Si atoms (the Si atomic radius is approximately 0.146 nm, which is smaller than the Ti atomic radius) into the TiC lattice may lead to the deviation of TiC from the standard diffraction peaks [76]. The higher the pyrolyzing temperature, the more Si atoms will diffuse into the TiC crystal lattice and cause larger TiC peak position shifts. Moreover, by changing the ratios of the raw materials, TiC crystals with different morphologies can be obtained, including octahedrons, truncated-octahedrons, and polyhedrals. These morphologies are mainly determined by the content of C during the reactions. Deficiency in C in the Si-Ti-C system will promote the accelerated growth of (111) facets, so perfect octahedrons will form. Upon increasing the C content, the formation of imperfect octahedrons will be favorable. They also demonstrated that the fusion agent CaF_2_ may influence the reaction process and change the morphology of TiC into a near-spherical morphology. The addition of CaF_2_ could change the solid-solid path between polycarbosilane and the metal mixture into a solid-liquid path. However, this solid-liquid path will introduce some impurities or defects onto the initializing (111) plane, so the growth rate of the (111) planes will be accelerated, and these particles will gradually grow into near-spherical shapes. Other fabrication processes, such as thermochemical reaction in acetone, can also yield TiC nanostructures. Yuan et al. fabricated TiC nanocrystal clusters on the surface of Ti particles, which are characterized in Figure 15c,d, and the application of TiC in electromagnetic wave absorption could also be obtained [120].

In nickel matrix, the reaction mechanism is also mainly related to the dissolution-precipitation process of TiC in the Ti-Ni-C ternary solution. The addition of TiC can also significantly enhance the anti-corrosion performance of Ni-base composites synthesized via selective laser melting [121]. Zhu et al. synthesized TiC in a 20 wt.% Ni-Ti-C system by combustion synthesis and explored the synthesis mechanism: under low temperatures, Ni-Ti solid-state diffusion started and formed Ti_2_Ni and Ni_3_Ti. Then, at 737–900 °C, not only Ti_2_Ni and Ni_3_Ti, but also NiTi and nonstoichiometric Ti_8_C_5_ were formed [122]. As the temperature further increased, Ni-Ti liquid formed, and then further C diffusion into the melting Ni-Ti liquid occurred, after which a Ni-Ti-C liquid solution formed. Finally, stoichiometric TiC was precipitated. They also found that the size of the spherical TiC particles increased as the heating rate increased. For instance, the size of TiC was approximately 1 µm (with the heating rate of 5 °C/min) and approximately 5 µm (at 80 °C/min).

Interestingly, a novel dendritic crystal structure of titanium carbide was reported for preparing TiC/Ti–Ni composites by Ma et al. [17]. Their original materials contained a mixed powder of Ti, Ni and TiC. By selective laser melting (SLM), the fine original TiC particles were transformed into in situ Ti_6_C_3.75_ dendrites. The gradual in situ formation of Ti_6_C_3.75_ dendrites from the original TiC particles is shown in Figure 15e. It mainly consists of two growing processes. The first is the epitaxial growth along the margin of the partly melted TiC particles, as shown in Figure 15f. Then, the fully melted TiC particles dissolve or precipitate. The further growth of the dendritic crystal is shown in Figure 15g. They also suggested that the corresponding influencing factor of the formation and growth of the Ti_6_C_3.75_ dendrites was mainly the thermal behavior of TiC particles within the molten pool. The diffusion effect of carbon was also important in this case.

The content of matrix can also exhibit an obvious effect for the Al, Cu and Fe matrixes; therefore, reactions in the Ni-Ti-C system perform similarly. Different from other reaction systems which force a high content of TiC in the Ni matrix, Liu et al. highlighted a novel technique that uses electrolytic nickel blocks as well as Ti and C powders to in situ synthesize low-TiC content reinforcements in molten Ni by a melting-casting method [123]. The final products were TiC and Ni, and the interfaces between TiC and Ni were quite clean. Here, Ni served as diluents and the reaction in this Ni-Ti-C system was simplified as Ti + C + cNi = TiC + cNi. Moreover, the Ni-Ti-C reaction in this case was mainly an eutectic reaction at the Ni-rich corner according to the phase diagram of the TiC–Ni system (20 vol.% TiC–Ni), and the TiC particles exhibited two shapes, with one being a large cubic shape and the other is a fine fibrous shape. It can be observed in Figure 15h that large cubic TiC represents the primary phase, while the relatively fine fibrous TiC represents the eutectic phase. This in situ TiC reinforced Ni matrix composite showed good mechanical properties, such as the relatively moderate yield strength and high hardness, ultimate tensile strength and transverse flexural strength.

Similar to the conclusion mentioned before, Ni can also influence the lattice parameter of TiC*_x_*, and Yang et al. emphasized that the TiC*_x_* lattice parameter decreased with increasing Ni content [124]. Their research also depended on the Ni-Ti-C system during the combustion synthesis. Because Ni can serve as a diluent in the Ni-Ti-C system, increasing the Ni content will obviously decrease the combustion synthesis temperature. It is known that a lower combustion temperature will decrease the atom diffusion rate, while a higher Ni content will also increase the diffusion distance, so the diffusion of atoms will be prevented, which is not favorable for compositional homogenization. Moreover, C can also diffuse into the crystal lattice of Ni, and the higher the Ni content, the more C atoms will diffuse into the Ni crystal lattice. Therefore, with the increase of the Ni content, the growth of TiC*_x_* will gradually become deficient in C, and the stoichiometric ratio of the TiC*_x_* will decrease.

This analysis based on atom diffusion also coincides with other reaction systems, while the influence of the matrix element may produce little difference. However, we can find that no matter whether Al, Cu, Fe, Ni, Si, or another element is used in the alloying matrix, the reactions of Ti and C in the matrixes can all be seen as dissolution-precipitation processes (for most of the reactions produced by combustion synthesis). The growth of TiC*_x_* always occurs in the melts, and the structures, stoichiometric ratios, sizes and morphologies of TiC*_x_* are sensitive to the growth kinetics and mechanisms and thermal and mass transportation in the melt under high reaction temperatures. For instance, the dissolution and diffusion of C atoms seems to be a pivotal procedure, which controls the growth behavior of the precipitated TiC*_x_* particles. On account of the intrinsic crystal structures as well as the specific external growth conditions in melt environments, the final morphologies of titanium carbides directly are reflected and will be different from each other. The following various properties will endow those titanium carbides with irreplaceable roles in mechanical application areas. Not only can the properties serve as refining agents to regulate and control the solidification structure or influence the precipitated phases, but they can also enhance the strength and ductility of the alloy. However, the inherent structural instability may restrict the applications of these composites, so more studies should be done to improve the structural instability and simplify the synthesis methods as much as possible. The specific measures may include the addition of some solution elements mentioned in the former chapter or novel fabrication, controlling and modification methods. Generally, the fundamental researches and application researches of titanium carbides will never be standstill.

### 5.2. Other Chemical Reaction Methods to Synthesize Titanium Carbides Particles

Moreover, reactions between Ti and C are not limited to Ti or C powders, but instead, various titanium and carbon sources are available. Chemical synthesis methods can be seen as an effective way to obtain more nanoscale TiC.

**Figure 16:**
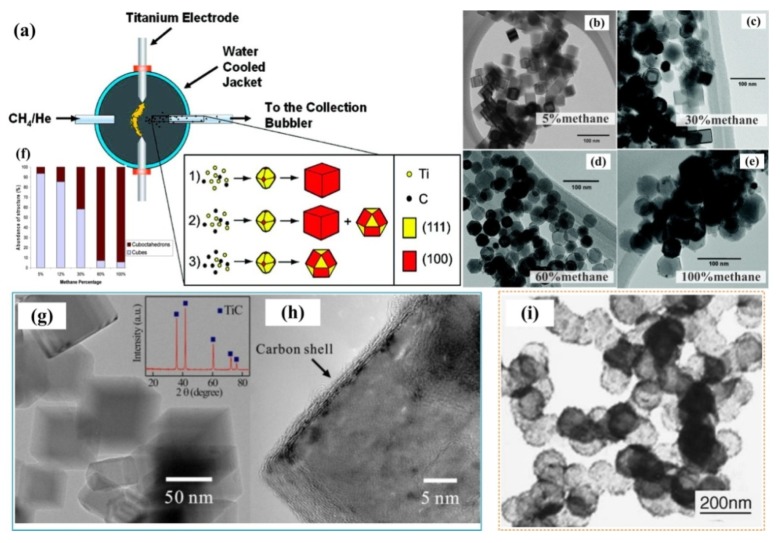
(**a**) Principle components of the arc discharge vessel and the various nanoparticle morphologies produced with different carbon concentrations. (**b**–**e**) TEM images of cube- and cuboctahedron-shaped nanoparticles synthesized with (**b**) 5%, (**c**) 30%, (**d**) 60% and (**e**) 100% methane. (**f**) Bar graph displaying the abundances of different morphologies synthesized with varying methane concentrations. It can be seen that at low methane concentrations, cubes were dominant, while at high methane concentrations, cuboctahedrons were dominant. With permission from Reference [41], copyright (2010) American Chemical Society; (**g**) TEM images of the TiC nanocubes and (**h**) the magnified detail of a cubic core-shell structure, with permission from Reference [125], copyright Elsevier, 2011; (**i**) TiC hollow spheres prepared at 400 °C. With permission from Reference [27], copyright Elsevier, 2004.

Grove et al. [41] successfully synthesized titanium carbide nanoparticles via an arc discharge method associated with flowing methane, and plasma was generated from an arc discharge between two titanium electrodes, as shown in Figure 16a–f. The morphology of the TiC nanoparticles can be regulated and controlled by changing the content of methane. Under a low methane supersaturation level, the growth rate of TiC [111] is quicker than that of the TiC [100], and the nanoparticles finally form cubic nanoparticles with six (100) facets; In contrast, under a high methane supersaturation level, cuboctahedral TiC particles with 14 facets (including 8 (111) facets and 6 (100) facets) will be dominant. In their studies, both cubes and the cuboctahedrons were formed from a truncated octahedron seed crystal with a face-centered cubic crystal structure, and the divergence of the shapes came from different growth rates of the (100) facet and (111) facet under different carbon supersaturation levels. Thie controlled growth of the stabilized TiC nanoparticles can potentially be applied in the fields of ceramic processing or chemistry catalysis.

Similarly, Meng et al. synthesized TiC nanocubes by arc-discharging a Ti target ingot in ethanol atmosphere [125]. As shown in Figure 16g and magnified in detail in Figure 16h, the as-synthesized TiC exhibited a cubic morphology with a size distribution varying from 5 nm to 20 nm. Obviously, it can be seen from the cube corner in Figure 16h that the TiC nanocubes were covered by a 2 nm-thick carbon shell. Nanocubic core-shell/TiC paraffin composites with proper mass ratios show prospective microwave absorption properties. Therefore, it can be concluded that novel structures such as carbon coated onto nanocubes seem to indicate a new tendency to explore more special properties such as anti-reflective microwave absorption properties.

Other hollow sphere morphologies of TiC have also been reported from the reaction between carbon nanotubes, TiCl_4_ and sodium at low temperature. Their reaction can be described as follows [27]:

TiCl_4_ (liquid TiCl_4_) + C (CNTs) + 4Na → TiC + 4NaCl (350–450 °C) (6)

The nascent titanium came from the sodium reduction of TiCl_4_. By interacting with carbon nanotubes, a special hollow sphere structure of TiC with a 55 nm outer diameter and a wall width of 10 nm on average can be produced, as Figure 16i shows.

## 6. Overview and Outlook

Recently, titanium and its alloys and compounds have attracted more and more attention due to many fascinating properties [126]. Simultaneously, the studies based on their binary or ternary compounds are also prevailing. Here, titanium carbides nanomaterials with excellent comprehensive properties have been widely studied and utilized in light-weight manufacturing, microwave absorption, electromagnetic protection, energy conversion and catalyst areas, etc. The special performances and applications of nanomaterials are mainly dependent on their external characteristics, especially the growth sizes and morphologies. In general, special sizes and morphologies will endow titanium carbide nanomaterials with superior properties such as high specific surface areas or high structural stabilities for applications. In this summary, we pay attention to the developments and breakthroughs of titanium carbides nanomaterials in the past decades from the perspective of their growth sizes and morphologies.

As Figure 1 and Figure 2 suggests, according to the different morphological dimensions of titanium carbides, the materials were divided into three categories, including one-dimensional nanostructures (named 1D nanostructures, including nanowires, nanorods, nanofibers, nanotubes, etc.), two-dimensional nanosheets (named 2D nanostructures, mainly MXenes) and three-dimensional nanoparticles (named 3D nanostructures, including sphericities, octahedrons, truncated octahedrons, cubes, hexagonal structures, dendrites, terraces, etc.). The synthesis of 1D nanostructures mainly occurs via two growth mechanisms: vapor-liquid-solid (VLS) and vapor-solid (VS). Under these mechanisms, the 1D nanostructure can grow along a specific crystal direction with or without a catalyst at the top of the nanostructure, and the final growth morphologies may be different when produced by different reaction mechanisms. More interestingly, the 2D nanostructures show complicated growth mechanisms and morphologies. The 2D nanosheets are mainly synthesized by the etching-assisted exfoliation of ‘MAX’ phases, and the produced 2D ‘MXenes’ maintain hexagonal crystal structures. Through the control of the synthesis processes, bare nanosheets and layered accordion morphologies with different interplanar spacings and some CNTs/nanocarbon-spheres attached on the terminated surfaces can be obtained. The fantastic microwave absorption and electromagnetic shielding properties of 1D and 2D nanostructures are indeed related to their special morphologies—the relatively high specific surface areas and some multiple internal reflection structures. Additionally, energy conversion and catalysis effects can be realized according to their external characteristics. For some 3D nanoparticles synthesized by combustion synthesis, the dissolution-precipitation process is the main reaction mechanism. The corresponding growth mechanisms are mainly lateral stacking of the growth units (under a low carbon concentration) and 2D nucleation growth (under a high carbon concentration). Therefore, it can be seen that the content of carbon plays a pivotal role in all cases, and the stoichiometric ratios of the as-synthesized titanium carbides will significantly affect the final growth morphologies. The specific exposed crystal surfaces and shapes will vary according to different synthesis methods. The 3D nanoparticles can act as significant refinements and reinforcements for the alloying matrix to improve its mechanical properties. Some particles can also be utilized for chemical catalysis according to their preferential exposed surfaces.

In addition, both the intrinsic crystal structures and some external reaction conditions lead to distinct growth morphologies and final properties of titanium carbides. Therefore, on the basis of the crystal structure of titanium carbide, a series of controlling factors has been reported for the synthesis titanium carbides with various external morphologies. Obviously, the growth of 1D, 2D and 3D titanium carbides proceeds through complicated processes and produces uncertainties and susceptibilities to the external environment, which lead their growth morphologies to deviate from the equilibrium states and become hard to control. However, in the past several years, more and more studies have obtained desirable titanium carbide morphologies during synthesis processes or further etching and heat treatments. In general, investigations of reaction mechanisms during synthesis are favorable for exploring the nucleation and growth behaviors of titanium carbides. From the initial structure design to the further control of the structure during the fabrication processes, the special sizes and morphologies of titanium carbides, along with their desirable performances, can be well manipulated and meet with their varied applications.

Actually, the morphology control of titanium carbides is still a prospective research direction. On one hand, the synthesis reactions should be better revealed, especially their thermodynamic and kinetic processes. In consideration of thermal and mass transportation in the reaction system, especially the dissolution and diffusion of the carbon sources, the reactions can be predicted and intervened. On the other hand, by controlling the reaction and then manipulating the growth behaviors of titanium carbide nanomaterials, the final morphology of titanium carbides produced under different synthesis environments can be controlled. Based on the theoretical guidance and oriented synthesis, a series of thorough studies about the growth behaviors and morphology control could be performed through more innovative synthesis routes. Moreover, innovative synthesis methods characterized by high efficiency, better energy conservation and environment protection are favorable for precise growth morphology manipulation and endow the as-synthesized nanostructures with excellent properties.

Last but not least, to realize the brilliant prospects of these titanium carbide nanostructures, more fundamental work should be done to precisely control the external characteristics, including sizes and morphologies. In the foreseeable future, fundamental studies of titanium carbides will never be at a standstill. Methods of artificially intervening and controlling the crystal growth behaviors to obtain desirable nanostructures with different morphologies will gradually become mature and favorable to meet the growing demand from engineering and functional fields.

## Figures and Tables

**Table 1:** Some nanoscale one-dimensional titanium carbide.

Morphology	Size	Preparation Method	Carbide Source	Potentional Applications
Nanorods	Diameter 80 to 200 nm Length 1 to 3 μm	Biotemplate method	Cotton T-shirt	Composites reinforcements; Nanoelectromechanical systems [8]
Nanowires	Diameter approximately 300 nm Length several microns	Infiltrating and chloride-assisted carbothermal reduction	Phenolic resol	Enhance the emission current for field emission applications [7]
	Diameter 200–400 nm Length dozens micros	Chloride-assisted carbothermal reaction	Sucrose	Electromagnetic wave absorbing [48]
Nanowhiskers	Diameter 300 nm to 2.5 μm	chloride-assisted carbothermal reduction method	Microcrystalline cellulose	Provide a new method and mechanism to synthesize TiC whiskers [10]
TiC/C Nanofibers	Diameter approximately 100 nm	Electrospinning	Polyvinylpyrrolidone	Supercapacitor [51]

**Table 2:** Summary of the Density functional theory (DFT) calculation results of titanium carbides MAX phases and MXenes from Naguib et al., with permission from Reference [61], copyright (2015) John Wiley and Sons.

Unit Cell Parameters (Å)			Volume Change
Formula	*a* = *b*	*c*	
Ti_3_AlC_2_ (Exp.)	3.080	18.415	
Ti_3_AlC_2_	3.058	18.554	–
Ti_3_C_2_	3.048	15.006	−19%
Ti_3_C_2_(OH)_2_	3.059	19.494	+5%
Ti_3_C_2_F_2_	3.019	21.541	+16%
